# Donut-like MOFs of copper/nicotinic acid and composite hydrogels with superior bioactivity for rh-bFGF delivering and skin wound healing

**DOI:** 10.1186/s12951-021-01014-z

**Published:** 2021-09-09

**Authors:** Tian-Long Wang, Zi-Fei Zhou, Jun-Feng Liu, Xiao-Dong Hou, Zhi Zhou, Yun-Lu Dai, Zhi-Yong Hou, Feng Chen, Long-Po Zheng

**Affiliations:** 1 Department of Orthopedics, Shanghai Tenth People’s Hospital, School of Medicine, Tongji University, 200072 Shanghai, China; 2 Shanghai Trauma Emergency Center, Shanghai, 200072 China; 3 Department of Orthopaedic Surgery, Third Hospital of Hebei Medical University, Shijiazhuang, 050051 China; 4 Cancer Centre, Faculty of Health Sciences, University of Macau, 999078 Macau SAR, China

**Keywords:** Wound healing, Tissue regeneration, Metal–organic frameworks, Copper, Hydrogel

## Abstract

**Background:**

Skin injury and the resultant defects are common clinical problems, and usually lead to chronic skin ulcers and even life-threatening diseases. Copper, an essential trace element of human body, has been reported to promote the regeneration of skin by stimulating proliferation of endothelial cell and enhance angiogenesis.

**Results:**

Herein, we have prepared a new donut-like metal–organic frameworks (MOF) of copper-nicotinic acid (CuNA) by a simple solvothermal reaction. The rough surface of CuNA is beneficial for loading/release basic fibroblast growth factor (bFGF). The CuNAs with/without bFGF are easily processed into a light-responsive composite hydrogel with GelMA, which not only show excellent mechanical properties, but also display superior biocompatibility, antibacterial ability and bioactivity. Moreover, in the in vivo full-thickness defect model of skin wound, the resultant CuNA-bFGF@GelMA hydrogels significantly accelerate the wound healing, by simultaneously inhibiting the inflammatory response, promoting the new blood vessels formation and the deposition of collagen and elastic fibers.

**Conclusions:**

Considering the superior biocompatibility, antibacterial ability and bioactivity, the CuNA and its composite light-responsive hydrogel system will be promising in the applications of skin and even other tissue regeneration.

**Graphic abstract:**

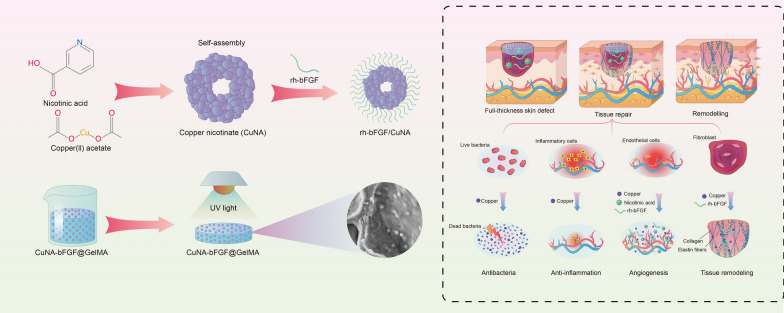

**Supplementary Information:**

The online version contains supplementary material available at 10.1186/s12951-021-01014-z.

## Background

Skin injury is usually caused by physical or chemical means [1], which may lead to chronic skin ulcers and threaten patient’s life, without timely medical intervention [2]. Wound healing is a complex biological process [3], which requires the coordination of a number of cell types to promote granulation tissue formation, and re-epithelialization. Therefore, many biomaterials with different functions have been developed to accelerate the tissue formation in the defect area.

Recently, hydrogels based biomaterials have attracted much attention, which can maintain a moist wound environment, prevent tissue dehydration and cell death, facilitate angiogenesis and collagen synthesis [4–6]. In particular, gelatin methacrylate (GelMA) is a promising hydrogel used for wound healing, due to the fact that it can mimic the properties of the extracellular matrix (ECM) such as high porosity, and predominant biocompatibility [7, 8]. As a derivative of gelatin, GelMA contains the arginine-glycine-aspartic acid (RGD) sequences and target motifs of matrix metalloproteinase (MMP) that support cell adhesion and remodeling [9–11]. In addition, GelMA possesses a majority of methacryloyl groups with a property of photo-crosslinking. It could undergo a process of polymerization initiated with free radicals that generate from the photo-initiator like lithium acylphosphinate salt (LAP) to form covalently cross-linked hydrogels after exposure to the particular light (e.g., UV) [12]. The resultant hydrogel displays biomimetic structure that favors cells proliferation and adhesion. However, GelMA has no other obvious bioactive properties, which limits its applications for wound healing. Although a majority of GelMA hydrogel dressings have doped with various drugs and ions for inhibiting infections, the unsustainable release, especially the initial burst release may lead to a passive healing process due to the biological toxicity of overdose [13].

Metal–organic frameworks (MOFs) are consisted with inorganic metal ions and organic ligands through coordination bonds, which are a class of coordination crystalline polymers with periodic structures [14, 15]. Due to the tunable chemical and physical properties, various MOFs based materials have been developed and used in the fields of chemistry, energy, and biomedicine [16]. Especially, the characteristics of MOFs provide an inspiration for developing new functional biomaterials, such as the MOFs constructed with bioactive metal ions and biomolecules with high biocompatibility. As one of the essential trace elements in the human body, copper has been demonstrated to facilitate the release of vascular endothelial growth factors (VEGFs) and cytokines via coordinating the expression of hypoxia-inducible factor (HIF-1a), which could promote the proliferation of endothelial cells and angiogenesis [17, 18]. In addition, copper ions could stimulate the expression of several important factors including MMP-1 and IL-8, which may favor the proliferation and extracellular matrix deposition of fibroblasts [19]. Moreover, copper ions play a crucial role in inhibiting inflammatory response, and exhibit excellent anti-bacterial ability [20, 21]. However, the sustained release of copper ions is critical for its in vivo application. MOFs constructed with copper ions and biomolecules could simultaneously solve these problems of sustained release of copper ions and biocompatibility of the organic ligands. Meanwhile, copper-based MOFs are easy to prepare the composite hydrogel materials, which helps to realize the gradient release of copper ions for promoting skin wound healing. In addition, recombinant human growth factors have shown great potential in promoting wound healing in the past few years. Among them, basic fibroblast growth factor (bFGF) plays an important role in wound healing for its biological effects, especially in stimulating the proliferation of fibroblasts and endothelial cells that give rise to granulation tissue, promoting the re-epithelialization and tissue remodeling [22].

**Scheme 1:**
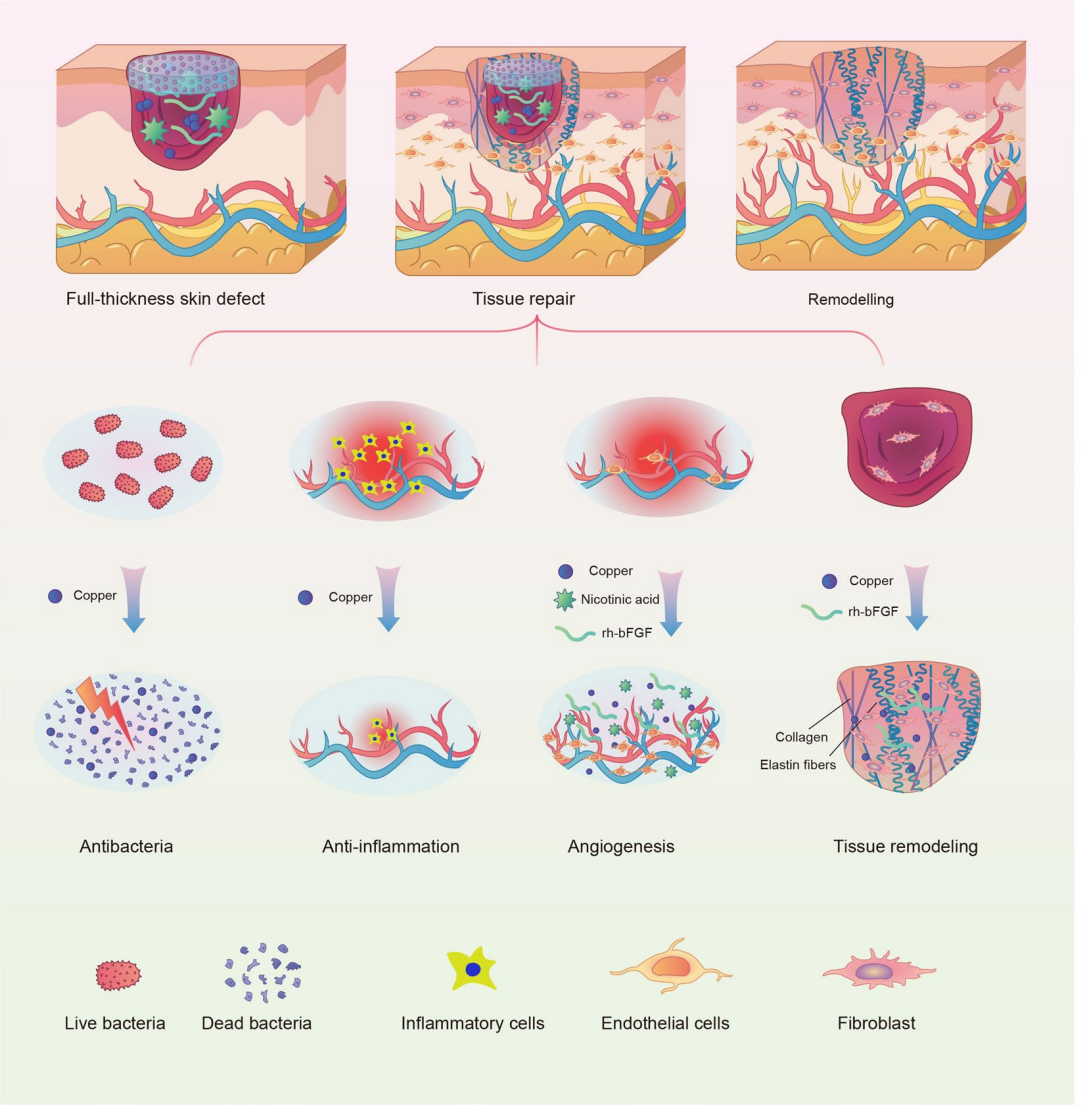
Illustration of the multifunctional CuNA-bFGF@GelMA hydrogel for accelerating the process of wound repair

In this study, we have firstly prepared a new copper-based MOF material (CuNA) with a donut-like structure, using a native biomolecules of nicotinic acid (NA) as organic ligand. CuNA have rough surface and could be used for loading and release of bFGF which can accelerate skin regeneration by promoting the proliferation of cells and angiogenesis [23–25]. Then, the CuNA with/without bFGF can be easily processing into light-responsive hydrogels after blending with GelMA. The resultant CuNA@GelMA shows excellent mechanical properties, such as flexibility and recoverability. Moreover, it not only displays outstanding antimicrobial activity against both Gram-positive and Gram-negative bacteria, but also shows high biocompatibility and bioactivity to promote cell migration and angiogenesis, due to the synergistic effects of copper and bFGF. As shown in Scheme 1, in the in vivo full-thickness defect model of skin wound, the resultant CuNA-bFGF@GelMA hydrogels significantly inhibited the inflammatory response, and promoted the new vessels formation and the deposition of collagen and elastic fibers, which greatly accelerated the wound healing process. This study strongly suggests that CuNA and its composite hydrogels possess multifunctional effects and hold a great potential for wound healing.

## Results

### Characterizations of CuNA and CuNA-bFGF

**Fig. 1:**
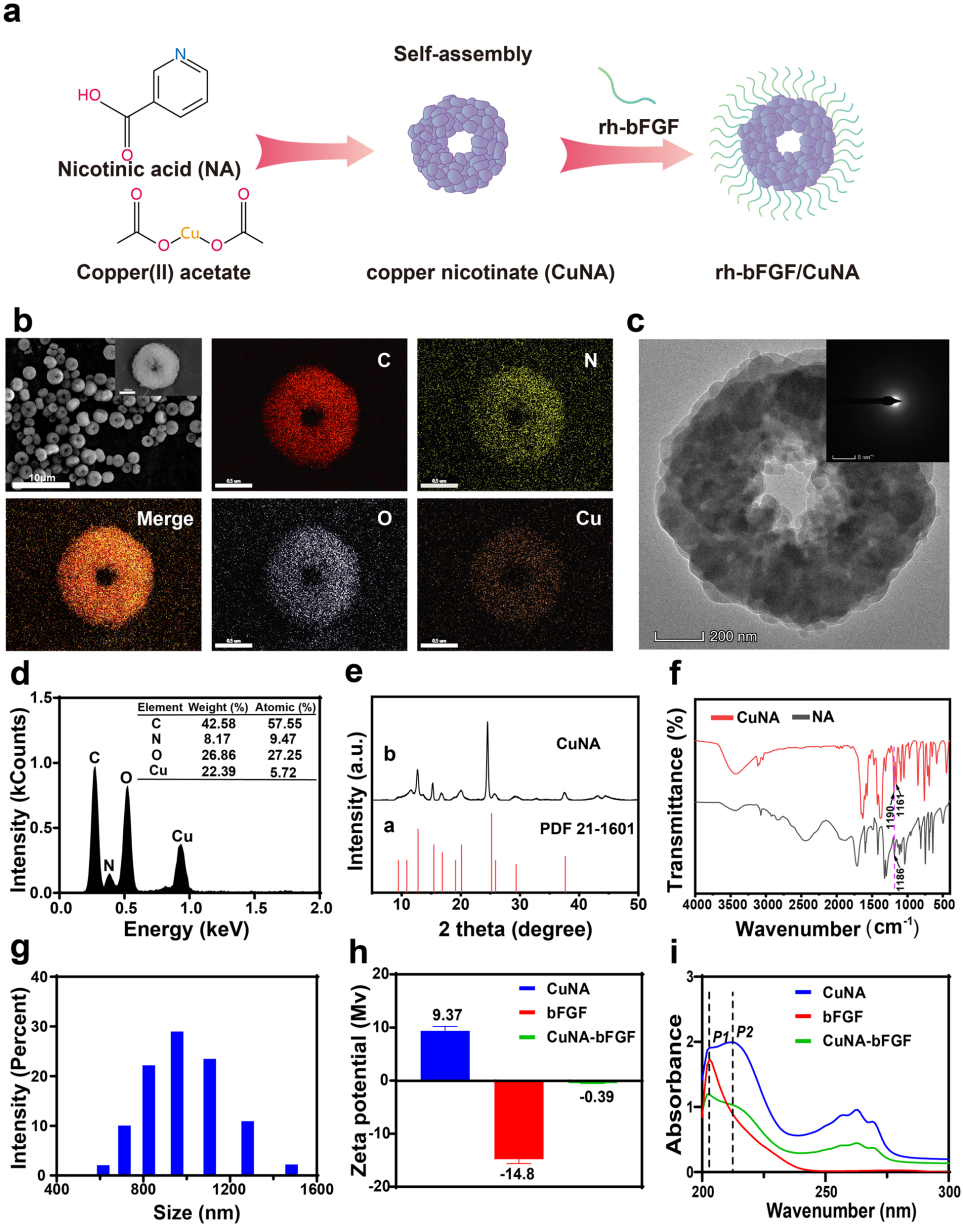
Characterization of CuNA prepared in ethylene glycol. (**a**) Schematic diagram of the self-assembly of CuNA and loading of bFGF. **b** SEM and elemental mapping images of CuNA. **c**, **d** TEM and EDS of CuNA. **e** The X-ray diffraction (XRD) patterns of CuNA. **f** FTIR of nicotinic acid and CuNA. **g** The size distribution of CuNA in distilled water. **h** Zeta potential of bFGF, CuNA and CuNA-bFGF. **i** UV–Vis of bFGF, CuNA and CuNA-bFGF

Schematic diagram of the self-assembling process and characterization of CuNA prepared in ethylene glycol are displayed in Fig. 1. The structures of CuNA prepared in ethylene glycol were observed by scanning electron microscopy (SEM) and transmission electron microscopy (TEM) (Fig. 1b, c). The SEM micrograph of CuNA clearly showed that the CuNA displayed a donut-like structure with relatively uniform micron-size (~ 1 μm). The distribution and ratio of elements among the CuNA were characterized by elemental mapping and Energy Dispersive X-Ray Spectroscopy (EDS) Energy (Fig. 1b, d). The atomic ratios of these elements are 57.55, 9.47, 27.26 and 5.72% for C, N, O and Cu elements, respectively. Meanwhile, the TEM micrographs of CuNA indicate that the proportion of ethylene glycol in the mixed reaction solution has obvious influence on the structure of CuNA (Fig. 1c and Additional file 1: Figure S1). There is a tendency that the donut structure is gradually formed with increasing the content of ethylene glycol. It is obvious that the donut-like structure has bigger surface area than the solid particles, which is beneficial for the modification and loading of bioactive substances. Therefore, CuNA particles prepared in ethylene glycol with donut–like structure have been selected to carry out the following studies. From TEM micrographs, it is clear to observe that CuNA is assembled by nanoparticles. The electron diffraction of selected area revealed that CuNA exhibit a typical crystal phase (Fig. 1c). Powder X-ray diffraction spectra (XRD) showed that the CuNA exhibited relative intensity of diffraction peaks at 12.7, 15.28, 16.7, 20.02, 24.52, 25.88, 29.16 and 37.44° with little deviations, which can be assigned to the standards (JCPDS card no. 87-1526) (Fig. 1e). In the Fourier Transform Infrared Spectrum (FTIR) of NA, the peaks appeared at 1186 cm^-1^ is assigned to C–O (COO–) stretching. Meanwhile, this C–O (COO–) stretching peak at 1186 cm^-1^ was splitted to two peaks (1190 and 1161 cm^-1^) at the FTIR spectrum of CuNA, which indicates the strongly interaction of Cu and NA, and confirms the successful preparation of CuNA MOFs (Fig. 1f).

More results of characterization have also been given in Fig. 1. According to the results of dynamic light scattering (DLS) measurements (Fig. 1g), CuNA particles exhibit an average diameter of ~ 980.8 nm, and Zeta-potential of CuNA is approximately + 9.37 mV. Then, the Zeta-potential value of CuNA increases to − 0.39 mV after loading with bFGF (Fig. 1h, Additional file 1: Figure S2), which indicated that the bFGF has been loaded onto CuNA by a facile electrostatic adsorption. In the spectrum of bFGF, the only absorption peak at ~ 202 nm is observed, which can be affirmed as a characteristic peak for bFGF. The spectrum of CuNA exhibits absorption peaks at ~ 202 nm (P1) and ~ 212 nm (P2), and it is also clear to see that the other peak at 212 nm is slightly higher than the peak at ~ 202nm. For the spectrum of CuNA-bFGF, the peak at 202 nm is the highest peak, and the peak at 212 nm is obviously decreased comparing with the spectrum of CuNA, which further confirm the loading of bFGF onto CuNA.

### Morphology of composite hydrogels

**Fig. 2:**
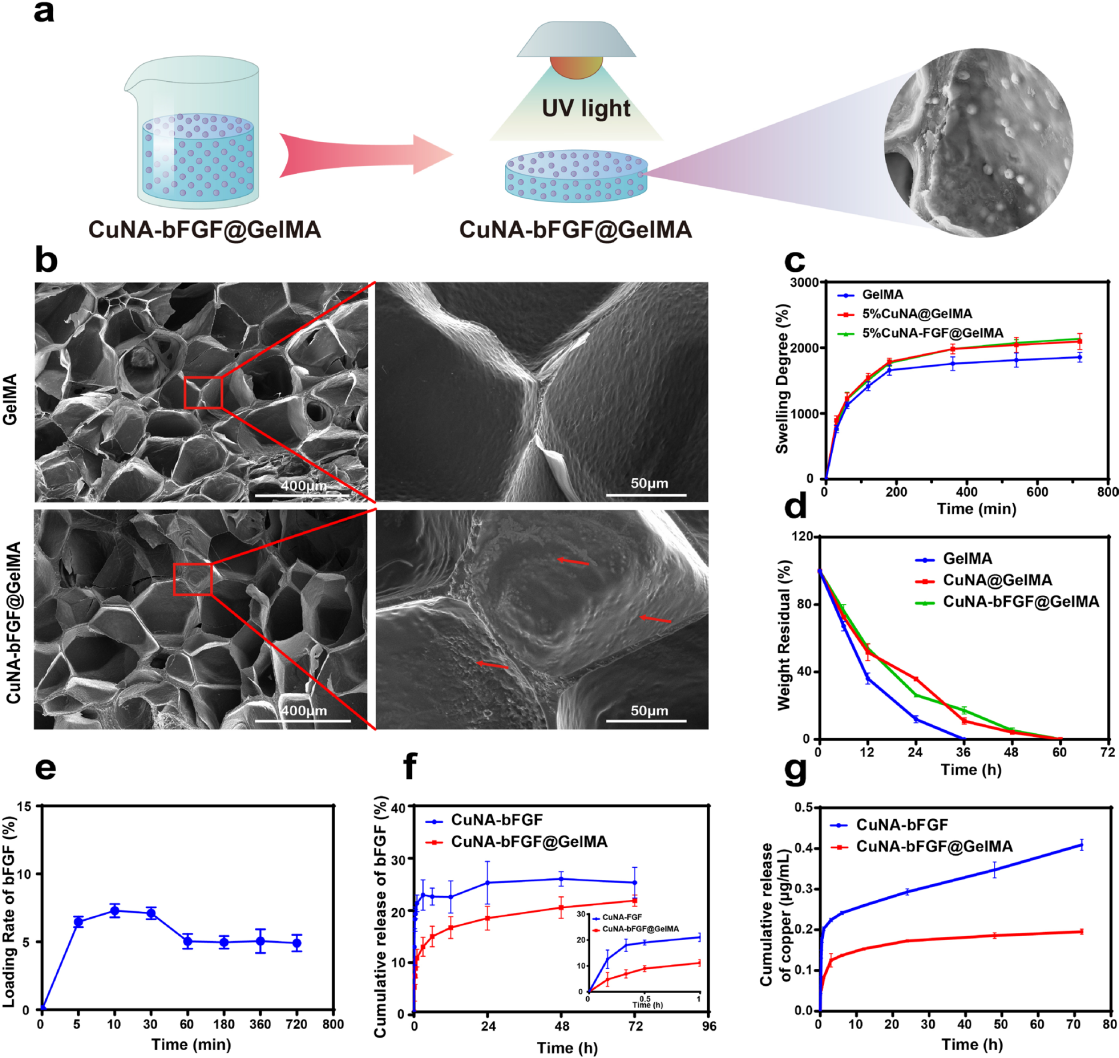
Characterization of GelMA composite hydrogels. (**a**) Scheme of the synthesis process. **b** Representative SEM image of the freeze-dried GelMA and CuNA-bFGF@GelMA. **c** Swelling kinetic curves of cross-linked GelMA at 37 °C in PBS (pH = 7.4). **d** Biodegradation property (in PBS + 1U/mL Collagenase solution) of different GelMAs for different time intervals at 37 °C. **e** The loading of bFGF on CuNA. **f** The release profile of bFGF from CuNA-bFGF and CuNA-bFGF@GelMA. **g** The release profile of copper from CuNA and CuNA-bFGF@GelMA. Data are expressed as mean ± SD (n = 3)

The GelMA based light-responsive hydrogels have been prepared as described in Fig. 2a. The microstructures of freeze-dried GelMA and CuNA-bFGF@GelMA hydrogels have been observed under SEM. As shown in Fig. 2b, both of samples show three-dimensional porous structure with an average pore size of ~ 200 μm. In the selected red cubic zones, the CuNA-bFGF embedded within the GelMA are obviously observed without changing the structures, which indicates a good compatibility between GelMA and CuNA particles. In addition, the chemical composition of CuNA-bFGF@GelMA has been analyzed with SEM and elemental mapping (Additional file 1: Figure S3). The C, N, P, S, Cu elements are well distributed in the composite hydrogels. The elements in composite hydrogels were further confirmed via X-ray Photoelectron Spectroscopy (XPS) as shown in Additional file 1: Figure S4. It is clear to see that the Cu2p spectra consists of Cu2p_3/2_ peak at 932.2 eV and Cu2p_1/2_ peak at 951.9 eV with slightly lower binding energy, which is consistent to the previous report [26].

### Swelling capacity and biodegradability of the composite hydrogels

The swelling property plays a key role in accelerating the wound-healing process by absorbing and retaining the wound exudates, which promotes cell proliferation and migration [27–29]. The satisfied swelling property of GelMA, CuNA@GelMA and CuNA-bFGF@GelMA were observed due to their porous network. The swelling ratio increased rapidly to the maximum within 1 h (Fig. 2c). All of the samples had an extremely high swelling ratio (> 1400%), indicating an excellent water absorption capacity. Notably, GelMA showed a higher maximum swelling ratio than GelMA composites, which may be due to the ionic strength increased in the swelling solution of GelMA composites. Consequently, osmotic pressure was higher in the swelling solution of GelMA composites, which limited the water absorption [30].

GelMA hydrogels are known to be degraded by collagenase enzymatic [31], and the degradation rate greatly influence the release behavior of ions or biomolecules. To investigate the effect of CuNA or CuNA-bFGF on the degradation of GelMAs, collagenase type II was used to evaluate the degradation properties of GelMA composite hydrogels. As shown in Fig. 2d, GelMA samples were completely degraded in 36 h, while the GelMA composites took nearly 60 h to degrade, which mainly due to the inhibition effect of copper ions on the collagenase enzymatic activity and the enhanced network of hydrogel via ionic cross-linking [32, 33].

### Loading and release characteristic of composite hydrogels

Figure 2e displays bFGF-loading properties of CuNA in phosphate buffered saline (PBS). When the CuNA particles are mixed in bFGF solution, CuNA can quickly adsorb the bFGF molecules, and then the adsorption capacity slightly decreases after the first 1 h to reach a relatively stable loading rate of ~ 4.97%. The result could be explained by the interaction between bFGF and CuNA. In the long-term soaking process, it is easily to understand that the Cu-NA complex on the surface of CuNA may partly dissolve, resulting in desorption of the adsorbed bFGF. With expending time, the surface of the CuNA becomes stable, which leads to a stable adsorption of bFGF.

Then, the release profile of bFGF from CuNA-bFGF@GelMA has been studied. As shown in the drug release curves, ~ 10% of bFGF loaded in CuNA-bFGF@GelMA has released into medium in the first 1 h, while, more than 20% of bFGF loaded in CuNA-bFGF has released (Fig. 2f). Then, the release properties of copper ions and NA molecules have also been studied. It is obvious to find that the release rate of copper ions from CuNA-bFGF@GelMA is much slower, comparing with that from CuNA-bFGF (Fig. 2g, Additional file 1: Figure S5). Moreover, the release of NA from CuNA-bFGF@GelMA is slower than that of CuNA-bFGF (Additional file 1: Figure S6). The sustained release properties of bFGF, copper ions and NA molecules from the composite hydrogel can be explained by their structure. CuNA-bFGF is embedded well within GelMA hydrogels (Fig. 2b), which serves as a barrier and avoid the initial burst release of these bioactive ingredients. In addition, copper could influence the enzymatic activities by bounding with sulfhydryl groups of proteins [33], which may inhibits the collagenase enzymatic activity and further diminished the degradation rate and drug release rates of the composite hydrogels. Meanwhile, copper ions can stabilize the structure of hydrogel via ionic cross-linking [32], which may also reduce the enzymatic degradation of GelMA composites. As a result, the release of bioactive ingredients from CuNA-bFGF@GelMA is a slower process in the PBS, comparing with that of the CuNA-bFGF particles in the same condition.

### Mechanical properties

**Fig. 3:**
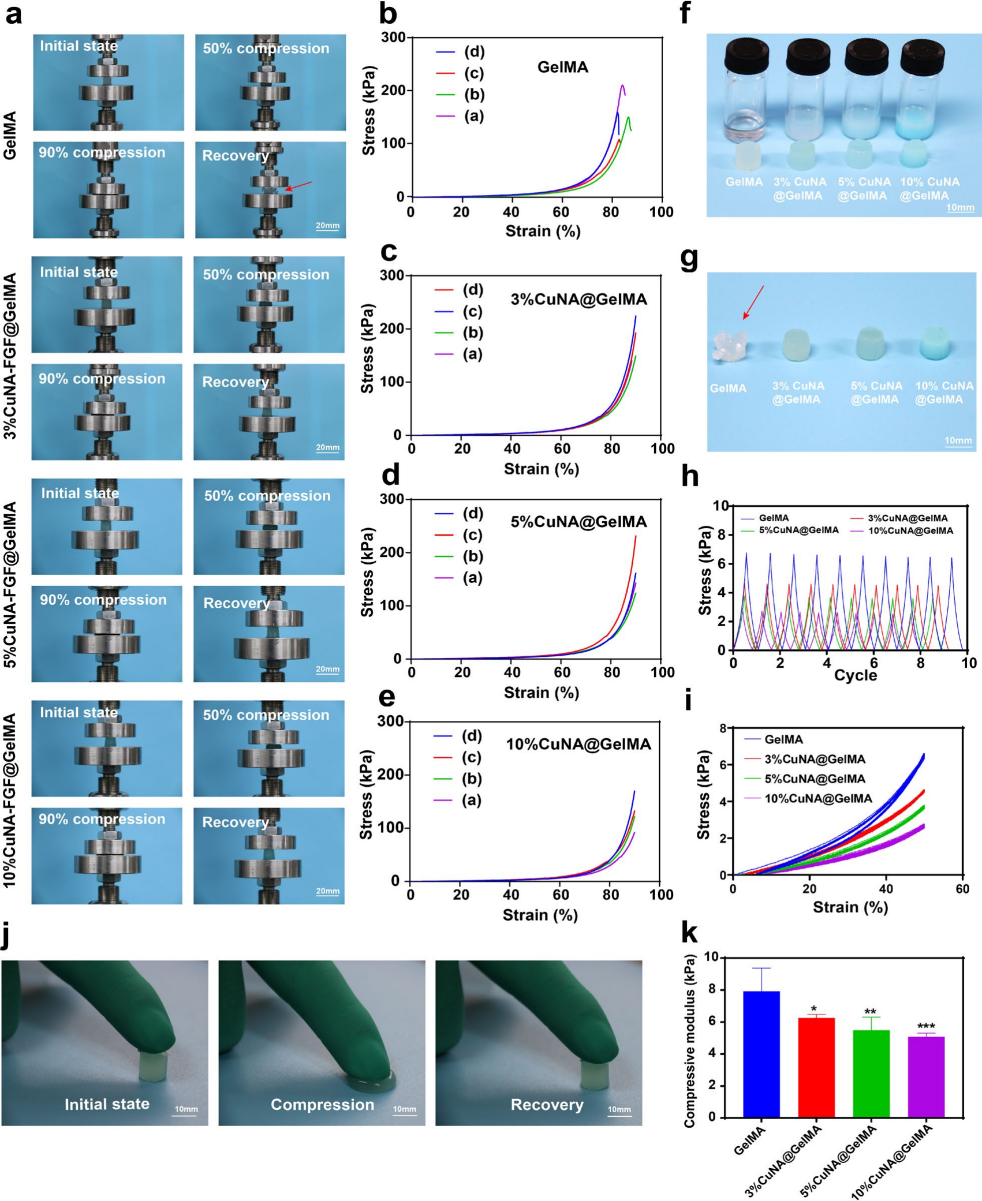
Mechanical characterization of the GelMA composite hydrogels. (**a**) Digital images of the compressive process (a. initial state; b. 50% compression; c. 90% compression; d. recovery). **b** Compressive stress–train curve of different GelMA composites. **f**, **g** Digital images of GelMA composite hydrogels before and after the compressive test. **h**, **i** The cyclic compression tests with ten cycles up to 50% strain. **j** Digital images of the real-time compressive process. **k** The compressive modulus of different GelMA composites. Data are expressed as mean ± SD (n = 4). (*p < 0.05, **<0.01, **<0.001 compared to GelMA)

The suitable mechanical properties of scaffolds including elasticity and recoverability are critically required in tissue engineering/repair, to promote the cell growth and tissue formation. Therefore, the mechanical properties of CuNA@GelMAs have been studied by compression tests. Digital images of GelMAs and CuNA@GelMAs samples with different content of CuNA in initial state, 50% strain, 90% strain and recovered samples after releasing pressure are shown in Fig. 3a. The related curves for mechanical analysis are given in Fig. 3b–e, which displayed an obvious trend that the elasticity of CuNA@GelMAs is much better than pure GelMA. The GelMA and CuNA@GelMAs samples in the initial state is shown in Fig. 3f, as well as the solution for preparing these samples. It is clear to see that these CuNA@GelMAs are not damaged when 90% strain has been applied to these samples, and can even recover to their initial state. However, the sample of pure GelMA group are fractured when 90% strain has been applied (Fig. 3g, red arrow). The results indicate that the incorporation of CuNA significantly enhanced the mechanical properties of GelMA. As shown in Fig. 3j, a set of real-time images of a compressing and recovering process of 5% CuNA@GelMAs indicate the excellent elasticity of CuNA@GelMAs. The compressive moduli calculated from the linear slope of the strain–stress curve shows decreasing values for GelMA, 3%, 5 and 10% CuNA@GelMAs respectively (Fig. 3k). Ten cycles of compress-release process at 50% strain showed much better recoverability in CuNA@GelMAs (Fig. 3h, i) than pure GelMA samples. The excellent mechanical properties of CuNA@GelMAs may be due to that the ionic cross-linking effect between copper ion and GelMA molecule which enhance the structure of composite hydrogels.

### Antibacterial activity in vitro

**Fig. 4:**
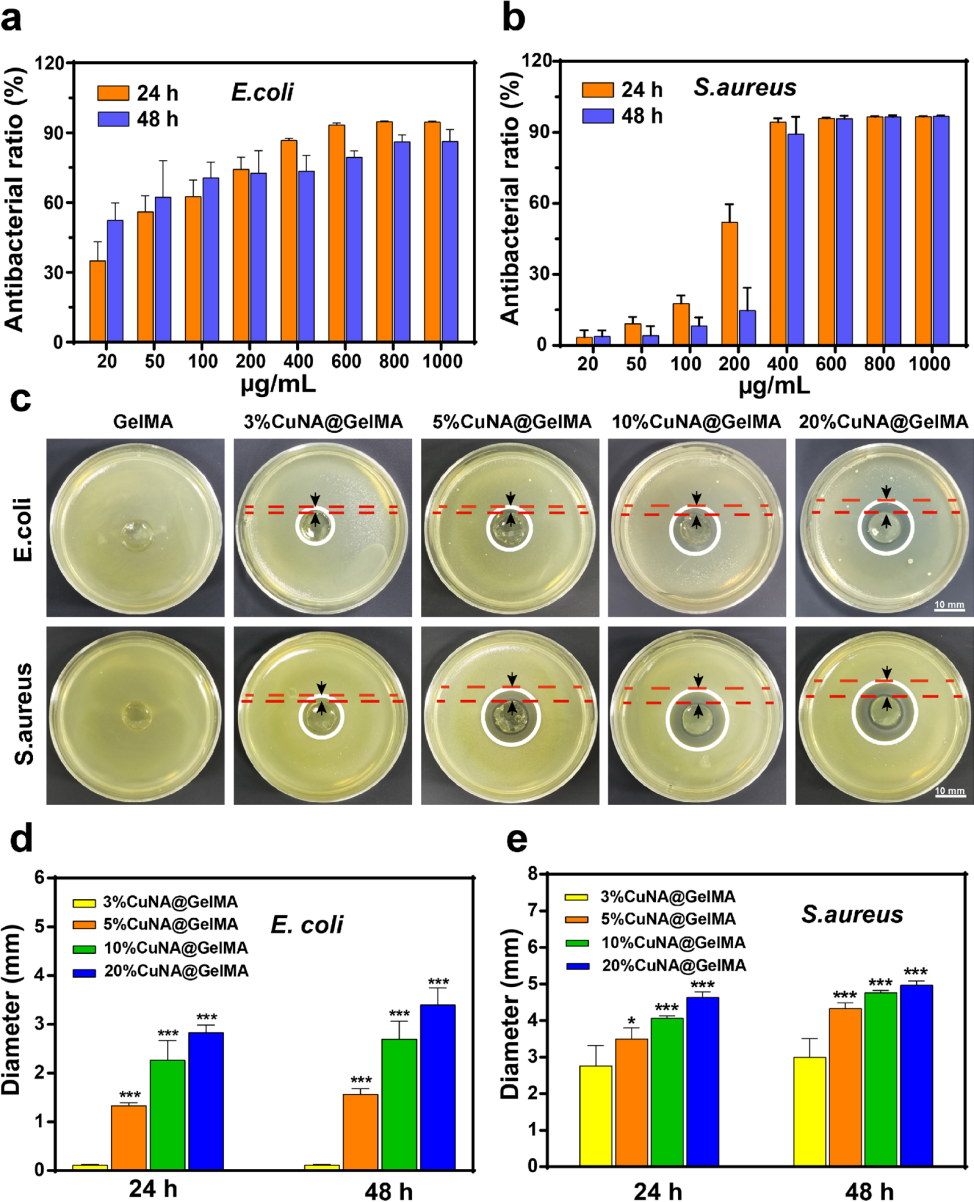
Antibacterial activities of different concentrations of CuNA and GelMA composite hydrogels (**a**, **b**) The antibacterial rate of different concentrations of CuNA against *E. coli* and *S. aureus* after 24 and 48 h contact time. **c** The inhibition zone of different divalent ions cross-linked GelMA hydrogels against *E. coli* and *S. aureus* photographed by the digital camera after 48 h. **d**, **e** Antibacterial rate of different GelMA composites is represented by the inhibitory zone diameter. Scale bar is 10 mm. Data are expressed as mean ± SD (n = 3). (*p < 0.05, **<0.01, **<0.001 compared to the 3% CuNA@GelMA)

The antibacterial activity is one of the most important properties for hydrogel dressings during wound healing [34]. The antibacterial capacity of the as-prepared materials has been evaluated in vitro with typical Gram-negative bacteria of *E. coli* and Gram-positive bacteria of *S. aureus.* In antibacterial activity assay, different concentrations of CuNA were incubated with each bacterium for 24 and 48 h. The antibacterial ratios against two types of bacteria were determined by the MTT assay shown in Fig. 4a and b. The results indicate that the antibacterial property of samples increase with increasing the concentration of CuNA. Notably, the antibacterial property of CuNA toward *S. aureus* is significantly improved when the concentration of CuNA reaches to 400 µg/mL. In addition, we notice that the antibacterial activity of CuNA with incubation time of 48 h is lower than the group with incubation time of 24 h at the concentration of 200 µg/mL. The results may be attributed to the adaption of *S. aureus* to copper stress and potentially also through modulation of cell wall stress responses [35]. In inhibition zone assay, CuNA@GelMAs showed a good antibacterial ability toward two types of bacteria compared to the control group of GelMA. Moreover, with increasing the content of CuNA in the composite hydrogels, a significant enhancement on antibacterial properties is observed. In addition, there is no obvious antibacterial activity found toward *E. coli* in 3% wt CuNA@GelMAs, while clear inhibition zone was found toward *S. aureus*, using the same concentration of GelMA composite (Fig. 4c), which may be due to that *S. aureus *is more sensitive to Cu ions than *E. coli*. Compared with the 3% CuNA@GelMA group, other groups with higher concentration of CuNA show a better performance in inhibition the growth of these two bacteria, with significant difference in the diameter of inhibition zones (*p* < 0.05, Fig. 4d and e), and the pure CuNA disks show excellent antibacterial ability toward these two bacteria with the biggest inhibitory circles (Additional file 1: Figure S7). It is well known that copper ions play an important role in preventing infection without using antibiotics. The previous studies have confirmed the antimicrobial activity of copper ions, and there are mainly two mechanisms, including membrane depolarization and reactive oxygen species (ROS) Generation [36, 37]. These results illustrated that CuNA@GelMA can effectively prohibit the proliferation bacteria, which is helpful for the effective antibacterial applications.

### Cell cytotoxicity and proliferation assessment

**Fig. 5:**
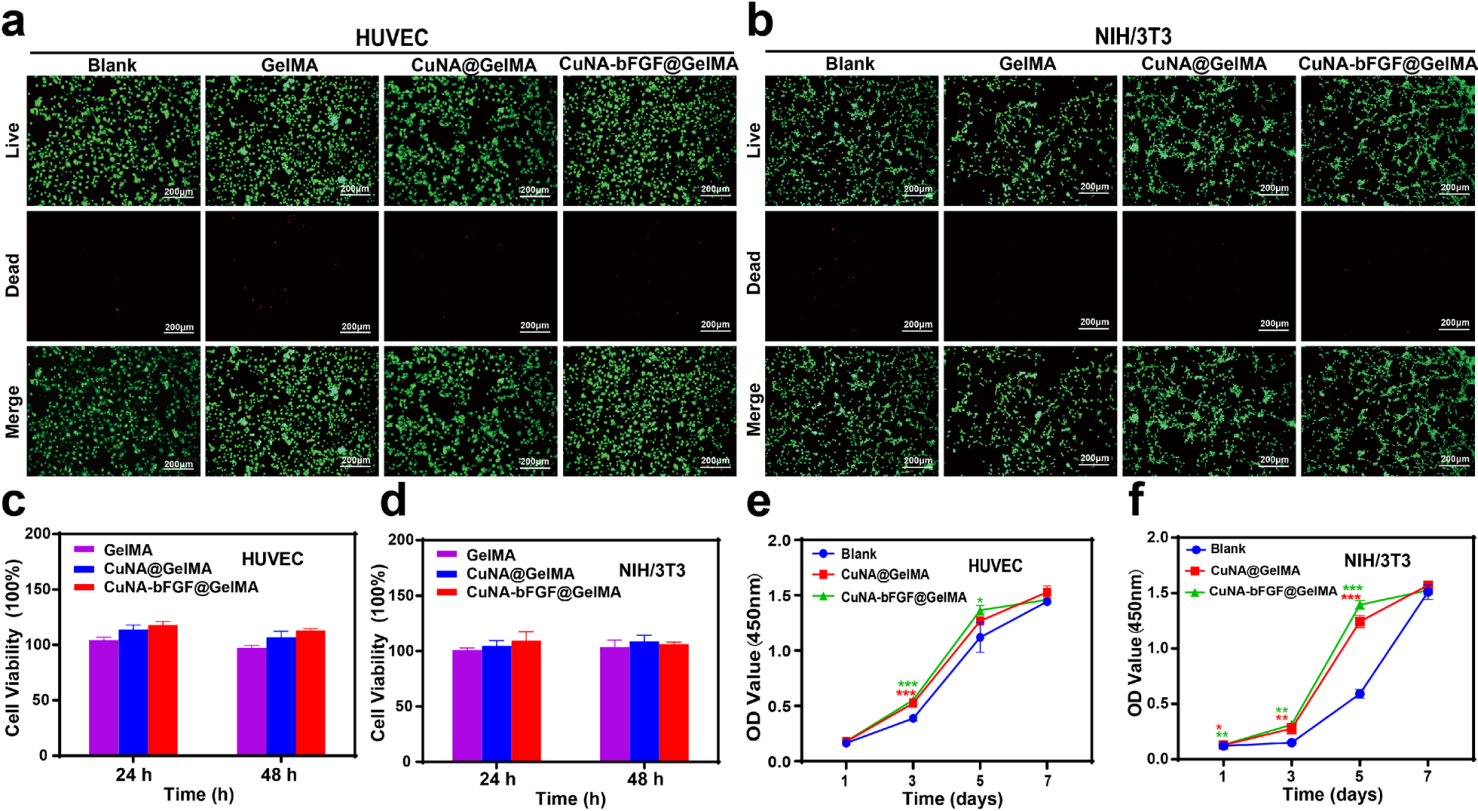
Biocompatibility evaluation of HUVEC and NIH/3T3 cells cultured in ionic extraction from 5% wt GelMA composite hydrogels (**a**, **b**) Live/dead staining of HUVEC and NIH/3T3 cells cultured in the complete medium with different ionic extraction for 24 h. Cell viability for HUVEC (**c**) and NIH/3T3 cells (**d**) was determined by CCK-8 method after cultured with different ionic extraction for 24 and 48 h. Cell proliferation assay for HUVEC (**e**) and NIH/3T3 cells (**f**) after cultured with different ionic extraction for 1, 3, 5, 7 days. Scale bar is 200 μm. Data are expressed as mean ± SD (n = 3). (*p < 0.05, **<0.01, **<0.001 compared to the blank)

To evaluate the biocompatibility of CuNA-bFGF@GelMA, a live/dead assay was performed to evaluate the viability human umbilical vein endothelial cells (HUVEC) and NIH/3T3 cells in vitro. If there is not particularly explain, CuNA@GelMA and CuNA-bFGF@GelMA refer to 5 wt% CuNA@GelMA and 5 wt% CuNA-bFGF@GelMA for all the follow-up experiments in cell viability studies. After being cultured in extraction for 24 and 48 h, the cell counting kit-8 (CCK-8) assay was performed to further evaluate the cell viability. As shown in Fig. 5a–d, the viability of cells culture with different extractions from GelMA, CuNA@GelMA and CuNA-bFGF@GelMA shows no significant difference, indicating a good biocompatibility of these samples. Moreover, the proliferation of cell in the extract mediums has also analyzed by CCK-8 assay, as shown in Fig. 5e and f. Especially on day 3, 5, there are significant differences between the composite hydrogels and the blank group, which may be resulted by the synergetic effect of copper and bFGF. However, no obvious difference is observed on day 7, which may be caused by the limited area for cell proliferation. Altogether, these results demonstrate that CuNA-bFGF@GelMA possesses excellent biocompatibility and can even promote cell proliferation in both HUVECs and fibroblasts.

### Cell morphology, migration and tubule formation activities

**Fig. 6:**
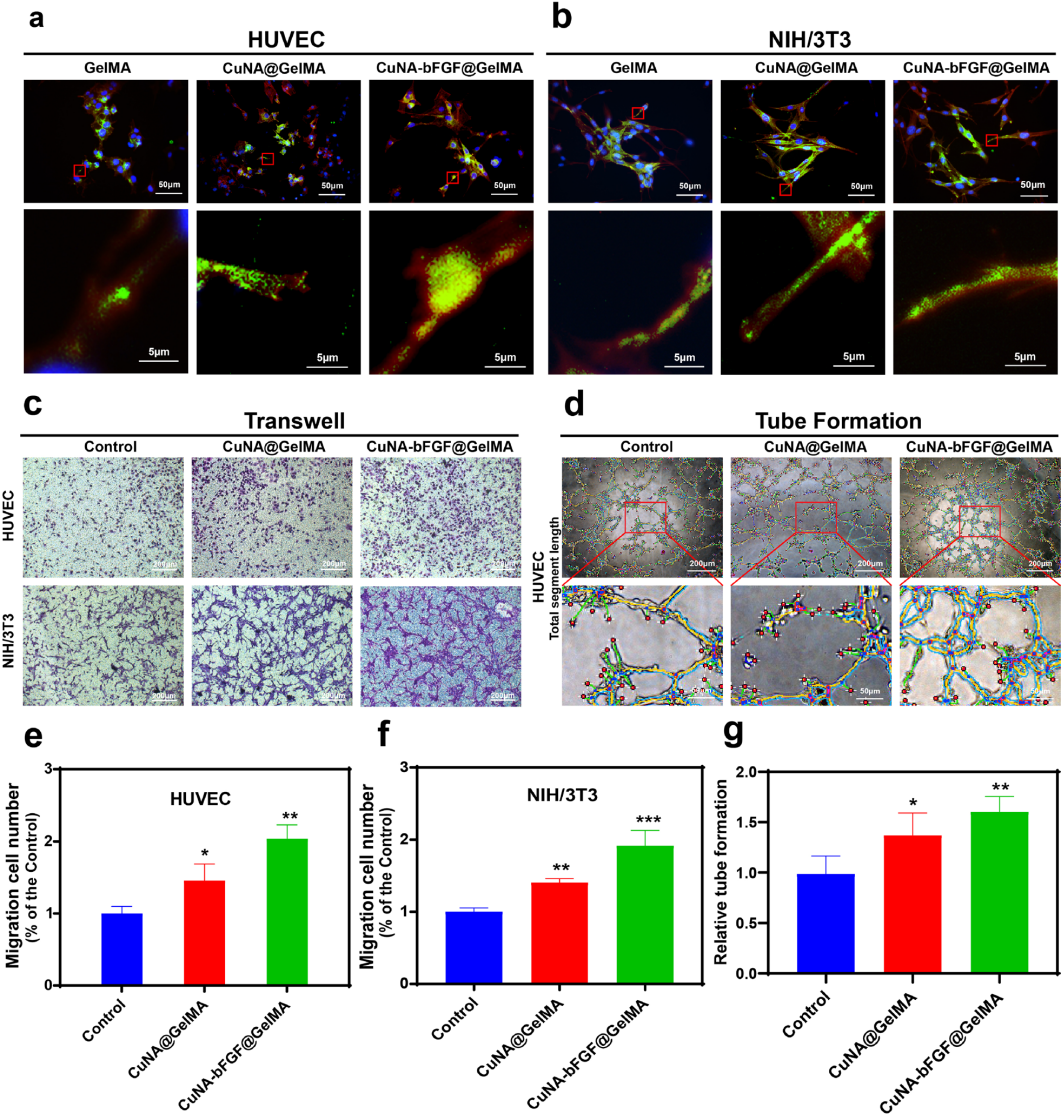
Cell attachment and spreading behavior, migration and tubule formation activities Cellular merged fluorescent images of HUVEC (**a**) and NIH/3T3 cells (**b**) on GelMA, CuNA@GelMA and CuNA-bFGF@GelMA hydrogels after 2 days post-seeding. F-actin (red), vinculin (green), nuclei (blue). **c** The migration evaluation of different ionic extraction on HUVEC and NIH/3T3 cells after incubation for 24 h by the transwell method. **d** The tube formation of HUVEC on matrigel after incubation with ionic extraction for 4 h. The migration number of HUVEC (**e**) and NIH/3T3 cells (**f**) compared to the control group. **g** The relative tube formation of HUVEC cultured in different ionic extraction. Data are expressed as mean ± SD (n = 3). (*p < 0.05, **<0.01, **<0.001 compared to the control)

The morphology of cells grown on GelMA composite was also observed under a confocal microscopy. From the fluorescent micrographs, it is clear to see that the cells can well spread on the pure GelMA and GelMA composites after 48 h of incubation (Fig. 6a, b), which indicate a well interaction between cells and materials. To investigate the migration ability of HUVEC and NIH/3T3 cells cultured in extraction medium of different samples, a transwell assay has been applied (Fig. 6c). The result shows that extraction mediums from GelMA composites can greatly increase the migration of HUVEC and NIH/3T3 cells, compared to the control group, as proven by a significantly higher migration ratio (p < 0.05, Fig. 6e, f). A tubule formation assay was conducted to evaluate the proangiogenic potential of HUVEC cells cultured in different extraction mediums (Fig. 6d). Comparing to the control group, the total segment length is significantly increased (*p* < 0.05, Fig. 6g) in GelMA composite groups. These results suggest that CuNA-bFGF@GelMA not only supported the migration of fibroblast and endothelial cells, but also promote the tubule formation of endothelial cells in vitro.

### In vivo wound closure measurement in full-thickness skin defect models

**Fig. 7:**
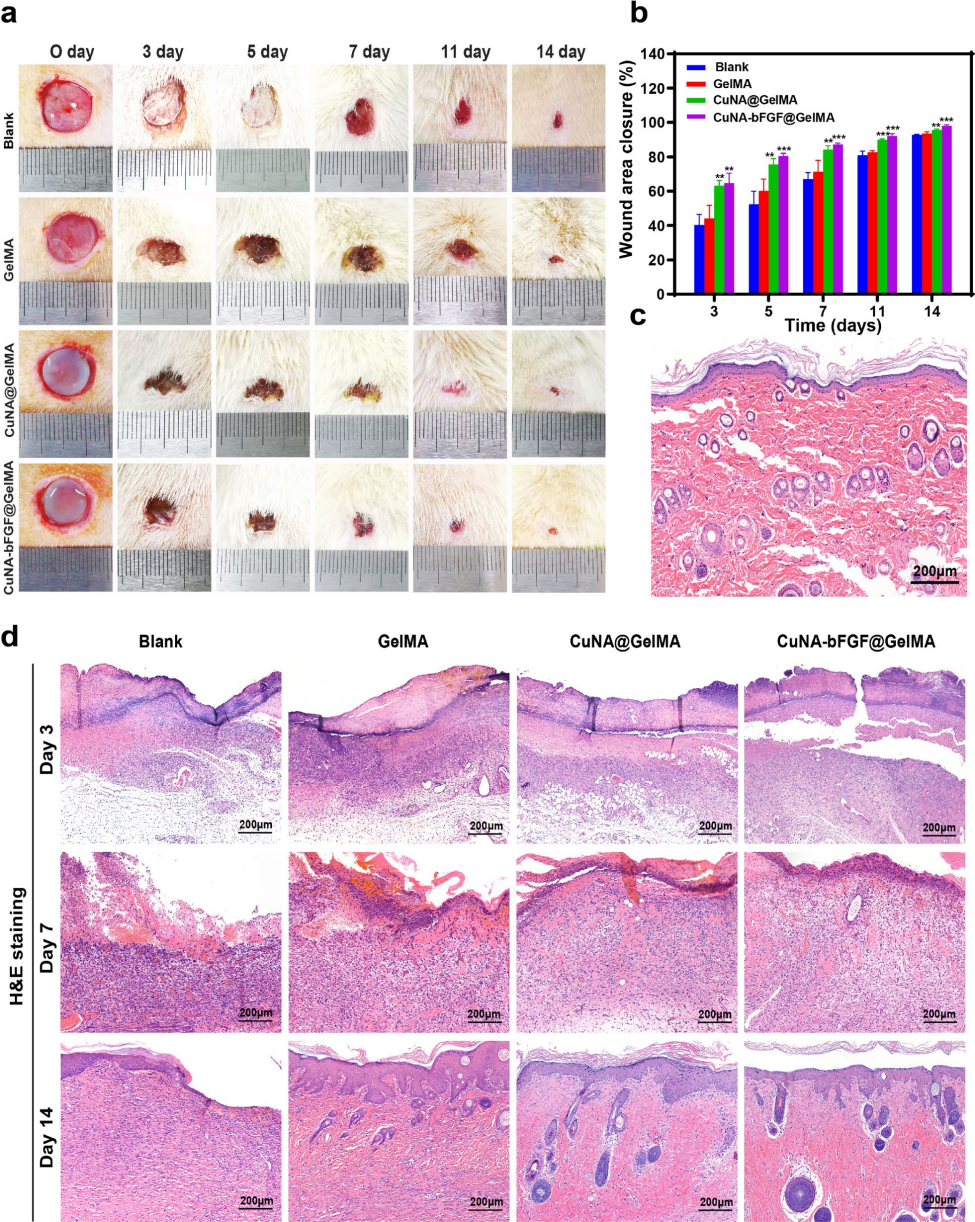
Wound closure and H&E staining (**a**) Digital images of full-thickness skin defects of SD rats treated with the GelMA, CuNA@GelMA and CuNA-bFGF@GelMA on day 0, 3, 5, 7, 11 and 14. **b** Wound closure percentages at different time points. **c** H&E staining for normal tissues. **d** H&E staining of wounds on day 3, 7 and 14. Scale bar is 200 μm. Data are expressed as mean ± SD (n = 3). (*p < 0.05, **<0.01, **<0.001 compared to the blank)

The wound-healing performance of 5% CuNA-bFGF@GelMAs as a potential wound dressing has been further investigated in a full-thickness skin defect model of rats. Digital images of the macroscopic appearance of wounds at different time points are shown in Fig. 7a. Though all the wounds shrank during each period, the percentage of wound closures in CuNA@GelMA and CuNA-bFGF@GelMA groups are significantly narrowed than other groups, as shown in Fig. 7b. Especially in CuNA-bFGF@GelMA group, a remarkable reduction in wound size is found at each period through the whole healing process, indicating that a synergistic effect of copper and bFGF accelerate the wound healing process.

### Histopathologic evaluations

**Fig. 8:**
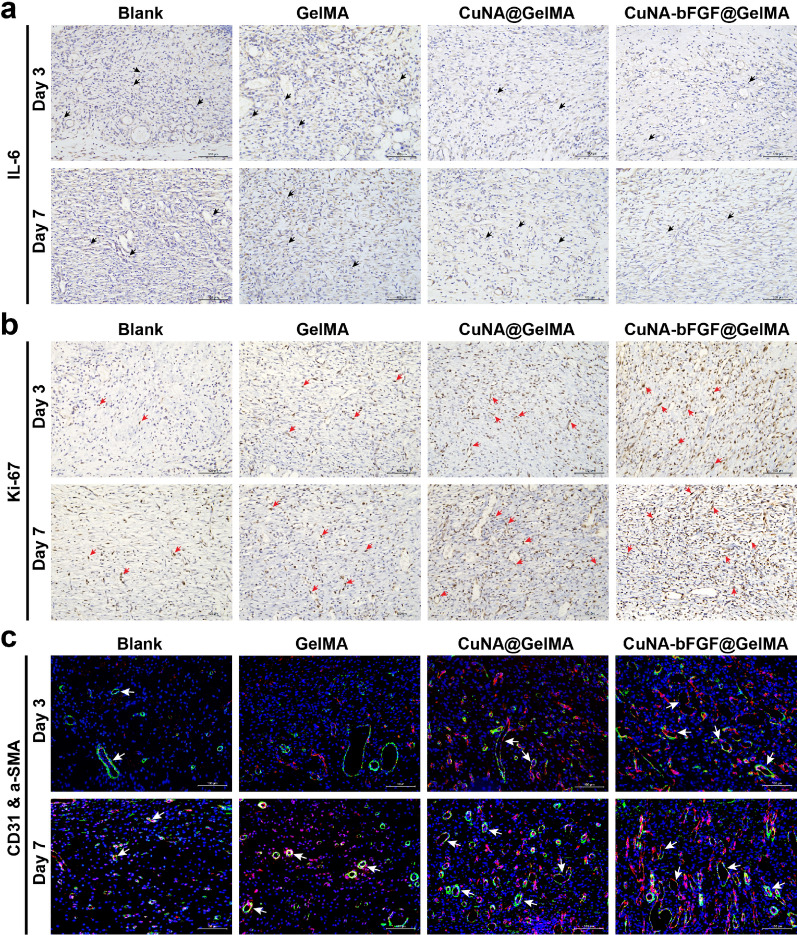
Histomorphological evaluation of wound tissues on day 3 and 7 (**a**, **b**) Immunohistochemical staining for pro-inflammatory factors of IL-6 and cellular proliferation marker of Ki-67. (IL-6 positive cells, black arrow; Ki-67 positive cells, red arrow). **c** Immunofluorescent staining of CD31 and α-SMA. Scale bar is 100 μm (new vessels marked by the white arrow; red: CD31; green: α-SMA; blue: nucleus)

Histological analysis of wounds was performed on day 3, 7, and 14 to evaluate the impact of CuAN-bFGF@GelMA on wound healing. H&E and Masson’s trichrome staining was conducted after 3, 7 and 14 days after treatment. As shown in Fig. 7d, although wounds in the blank and GelMA groups form the basic structure of epithelium and dermis, tissue is still under a healing process without hair follicles found. However, the CuNA@GelMA and CuNA-bFGF@GelMA groups show more regular epithelium, new blood vessels, and mild inflammatory responses. Especially the H&E staining of the CuNA-bFGF@GelMA group is closer to the normal tissue **(**Fig. 7c), indicating an excellent wound healing performance. To further evaluate the inflammation degrees of wounds in these groups, immunohistochemical staining has been applied to analyze the expression of interleukin 6 (IL-6) as the pro-inflammatory cytokines on day 3 and 7. As shown in Fig. 8a, the blank and GelMA groups express more IL-6 than the other two groups on day 3, while the excessive inflammation may encumber wound healing [38].

Ki-67 positive nuclei represent the re-epithelialization process. Figure 8b shows that a small number of Ki-67 positive nuclei are observed in the wound samples of the blank and GelMA groups, while progressively distribute in the newly formed tissue filling the central wound in CuNA@GelMA and CuNA-bFGF@GelMA groups. The higher expression of Ki67 marker is observed in GelMA composite groups than control groups, and the CuNA-bFGF@GelMA group exhibits the highest level among all the groups. These results indicate that the CuNA-bFGF@GelMA group can promote cell proliferation in the process of skin tissue repair.

Immunofluorescence staining of CD31 and a-SMA for mature blood vessels was also performed on day 3 and 7, to investigate the angiogenesis response of the composite hydrogels. As shown in Fig. 8c, GelMA composite groups show the stronger positive expression of α-SMA and CD31, comparing with the other two groups. Especially, the CuNA-bFGF@GelMA group show the strongest positive expression on both day 3 and 7. In addition, CD34 staining was also performed on day 3 and 7, to observe the newly vessel density which is defined as the number of CD34 positive cells. As shown in Additional file 1: Figure S8, the results show a better angiogenesis response in the CuNA-bFGF@GelMA group, which is consistent with the results of immunofluorescence staining of CD31 and a-SMA.

**Fig. 9:**
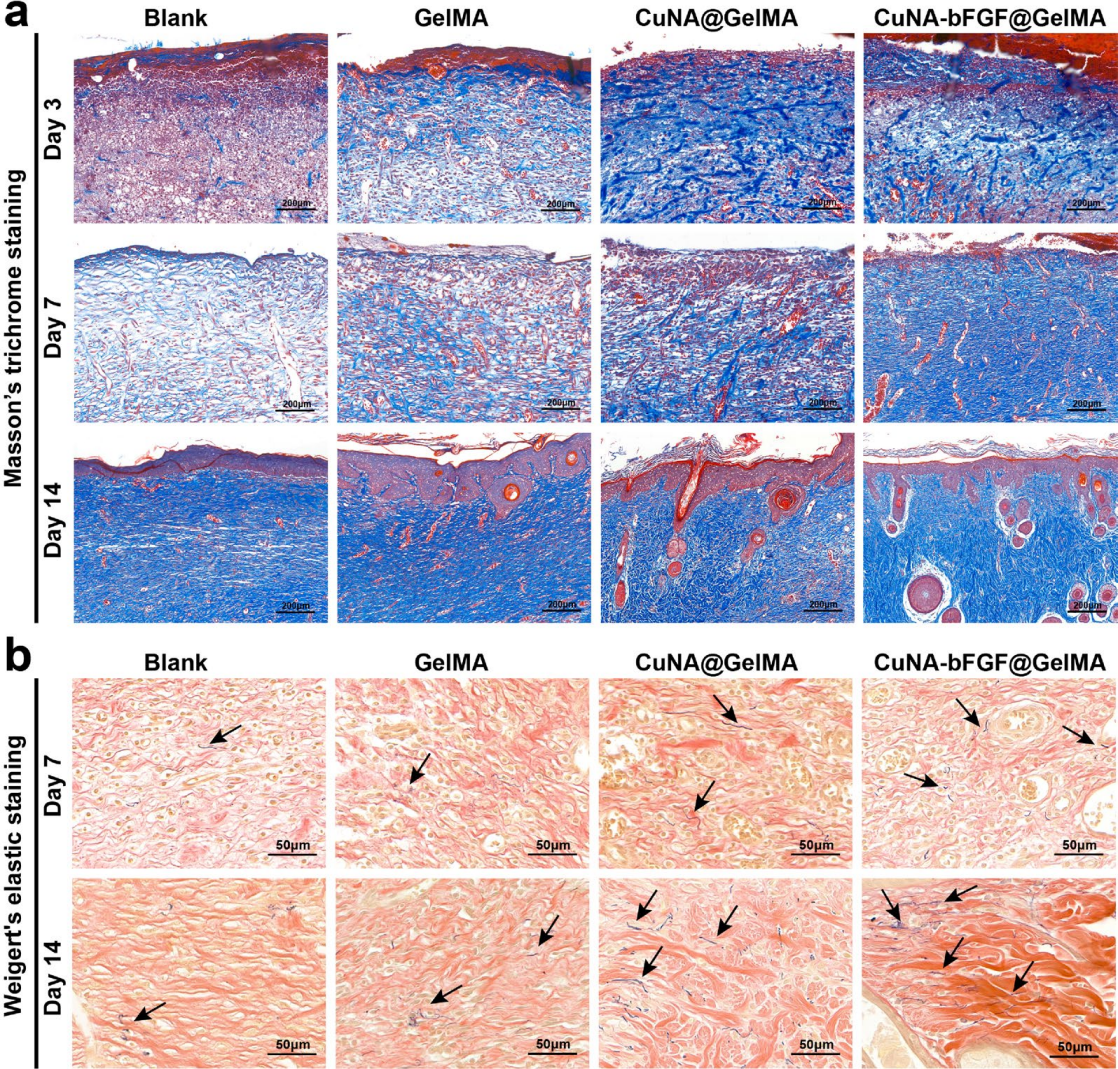
Masson’s trichrome staining and Weigert’s elastic staining (**a**) Masson’s trichrome staining was performed on day 3, 7, and 14. **b** Weigert’s elastic staining on day 7 and 14. (elastic fibers, black arrow)

It is reported that collagen formation is due to the massive proliferation of fibroblasts [39]. Collagen deposition in the defect areas of different groups has also been investigated in this study. The Masson’s trichrome staining displays that much more collagen deposition forms in the CuNA-bFGF@GelMA group (Fig. 9a), which is similar to the normal tissue (Additional file 1: Figure S9), indicating the efficiency of CuNA-bFGF@GelMA on promoting cells proliferation. To further evaluate the wound healing process, Weigert’s elastic staining was performed on day 7 and 14. As shown in Fig. 9b, the CuNA-bFGF@GelMA group shows more and thicker Weigert-positive fibers comparing with other groups, indicating the CuNA-bFGF@GelMA dressing benefited the formation of elastic fibers.

## Discussion

Among various hydrogel based biomaterials, GelMA with superiorities represents a promising approach in wound healing, tissue engineering and other biomedical applications. Conveniently, this kind of hydrogel is often cross-linked by light with photoinitiators at a mild condition such as room temperature and neutral PH. The polymerized time and space under the light greatly influence the crosslinking density and final structures of hydrogels, which provides an ideal strategy to fabricate GelMA hydrogels with controlled crosslinking density and structures, such as different 3D structure, some unique patterns or morphologies [7, 40]. What’s more, it is feasible to incorporate the functional nanomaterials into GelMA to improve the physical, chemical, and mechanical properties of hydrogels. For example, some particular growth factors and bioactive ingredients are necessary for the proliferation of endothelial cells and fibroblasts which play a key role in angiogenesis and collagen deposition to accelerate wound healing [12, 41].

Usually, bioactive molecules and functional nanomaterials are easily blended into GelMA to obtain the composite hydrogels for promoting tissue regeneration. By this way, some efforts to prepare functionalized GelMA have been made to enhance the antibacterial capacity and bioactivity in angiogenesis and cell proliferation [42–44]. In previous studies, copper ions have been confirmed to have the great potential in promote cell angiogenesis [45, 46], and copper-doped hydrogel had been fabricated for tissue engineering [47]. However, copper ions which quickly released from the hydrogel may limit the effective time, and the concentrated release of copper ions can probably cause toxicity. Meanwhile, some studies have reported that metal-based organic complexes may display better biological properties compared to the pure metal ions [48–50]. Therefore, the copper-based organic frameworks synthesized with copper ions and NA molecules could be an ideal strategy to achieve the biological effects of copper in promoting tissue repair. In addition, comparing with copper ions, copper-based organic frameworks could be a carrier to loading bioactive molecules, such as bFGF which plays an important role for its biological effects in skin regeneration and other tissue repair areas. Previous studies have shown the effectiveness of bFGF in the management of pressure ulcers [51], and some studies indicated that bFGF-loaded hydrogels could accelerate the wound healing process [52, 53]. Significantly, strategy for loading growth factors onto CuNA can probably induce to a synergistic biological effect of copper ions and bFGF, which may further accelerate the wound healing process.

In this study, the GelMA composite hydrogel showed multifunction by incorporating with copper-based metal-organic frameworks of CuNA and bFGF-loaded CuNA. After exposure to particular light, GelMA undergo a process of polymerization to form covalently cross-linked hydrogels, and CuNA and bFGF-loaded CuNA could be packaged into the composite hydrogels. The obtained CuNA@GelMA showed excellent mechanical properties and water-uptake capacity. In this complex hydrogel, copper may induce a ionic cross-linked network in hydrogel which optimize the mechanical properties of the hydrogel via a complicated ligand-metal complexation [32]. Meanwhile, in the drug release experiments, compared to CuNA-bFGF samples which are directly immersed in PBS, the release of bFGF and NA from CuNA-bFGF@GelMA exhibited a relative sustained process. The sustained release properties of CuNA-bFGF@GelMA can ensure an effective wound healing process [54]. In the biodegradation experiments, the GelMA composites took more time to degrade, which could also relieve the initial burst release of copper ions or growth factors.

The bioactivities of CuNA@GelMAs and CuNA-bFGF@GelMA are mainly represented on the effects of antibacterial properties and promoting cell proliferation. In antibacterial tests, CuNA@GelMAs show an excellent antibacterial ability, which can inhibit the infection and benefit the wound healing. In cell proliferation assay, the released copper ions and bFGF could stimulate the expression of many indispensable factors of cells, including VEGF, MMP-1, and IL-8. Due to the synergistic effects of copper ions and bFGF, the extraction medium of composite hydrogels, especially the CuNA-bFGF@GelMA can significantly promote cell proliferation, migration, and tubule formation.

In the studies of in vivo repair of full-thickness skin defect model for 14 days. the great potential of CuNA-bFGF@GelMA on accelerating the wound healing process is further confirmed. Comparing with other groups, the H&E staining of wounds in the CuNA-bFGF@GelMA group shows the best epithelialization effect. Immunohistochemical staining for IL-6 also indicates a mild inflammatory response in the CuNA-bFGF@GelMA group. Meanwhile, the immunofluorescence staining of CD31/α-SMA verify that the CuNA-bFGF@GelMA group exhibits the highest density of vessels on day 3 and 7. Immunohistochemical staining of CD34 on day 14 indicates that the most vessels in the CuNA-bFGF@GelMA group had grown mature, comparing to other groups. Meanwhile, the highest expression of Ki-67 positive nuclei demonstrated its excellent ability to promote cell proliferation. Furthermore, Masson’s trichrome staining and Wiegert’s elastic staining display that much more collagen and elastic fibers deposition are found in the CuNA-bFGF@GelMA group. Comparing with the control groups and normal skin, the CuNA-bFGF@GelMA group displays a uniform dermal collagen on day 14. Above results demonstrate that the CuNA-bFGF@GelMA dressings can efficiently promote the angiogenesis, collagen deposition and re-epithelialization process in the in vivo full-thickness skin defect model.

## Conclusions

In summary, we have demonstrated a simple strategy to prepare new donut-like MOF materials of CuNA, using copper ions and natural molecule of NA. Copper is an essential trace element of human body and can stimulate the expression of growth factors and cytokines, meanwhile, NA is a necessary vitamin B for the health of human. Therefore, CuNA not only solves the problem of low biocompatibility of traditional MOF materials, but also provides a new strategy for the controllable release of bioactive molecules. Moreover, the rough surface of the as-prepared CuNA is beneficial for loading/release growth factor of bFGF. Then, the CuNA with/without bFGF is blended with GelMA to obtain the light-responsive hydrogels. The resulted composite hydrogel of CuNA-bFGF@GelMA showed great water-uptake capacity, good biodegradation and relative sustained release behavior of bFGF and NA. Meanwhile, the composite hydrogels after freeze-drying displayed highly porous morphology which is suitable for cell growth. Furthermore, the composite hydrogels had displayed a great antibacterial ability and excellent mechanical properties of flexibility and recoverability. Significantly, we have clearly demonstrated that CuNA-bFGF@GelMA not only support the migration of fibroblast and endothelial cells, but also the tubule formation of endothelial cells in vitro, which may accelerate the wound healing process. Finally, in vivo experiments had proved that the CuNA-bFGF@GelMA (5% wt) could significantly accelerate the wound healing process in full-thickness skin defect models as evidenced by the reduction in the wound area, and the enhancement in angiogenesis, deposition of collagen and elastic fibers, and re-epithelialization. Considering the high performances in chemical/physical and biological characterizations, CuNA and the composite hydrogels of CuNA-bFGF@GelMA will be a promising biomaterial for wound healing.

## Methods

### Materials and regents

Nicotinic acid (NA), copper (II) acetate monohydrate, ethylene glycol were purchased from Aladdin Industrial Co. Ltd (Shanghai, China). Recombinant human basic fibroblast growth factor (bFGF) was purchased from Nanhai Longtime Pharmaceutical Co., Ltd. (Guang Dong, China). Gelatin methacryloyl (GelMA) was purchased from Engineering for Life (JiangSu, China). Chemicals for the preparation of Luria–Broth (LB) agar medium were purchased from Sangon Biotech Co. Ltd (Shanghai, China). BCA protein Assay kit, Live/Dead kit, Thiazolyl Blue Tetrazolium Bromide (MTT), 4% Paraformaldehyde Fix Solution, Dimethyl sulfoxide (DMSO), Crystal Violet Staining Solution, actin Tracker Red Rhodamine, DAPI were purchased from Beyotime (Shanghai, China). Phosphate buffered saline (PBS, pH 7.4), Fetal Bovine Serum (FBS), Dulbecco’s modified Eagle medium (DMEM), trypsin-EDTA and penicillin-streptomycin were purchased from Thermo Fisher Scientific (Waltham, MA, USA). Anti-vinculin-FITC antibody was purchased from Sigma-Aldrich (St Louis, MO, USA). 24-well Transwell (8 μm pore size), Matrigel Matrix were purchased from Corning (New York, USA).

### Synthesis of copper-based organic framework (CuNA)

NA (0.123 g, 1 mmol) and Copper (II) acetate monohydrate (0.1 g, 0.5 mmol) were dissolved in DI water and ethylene glycol solutions (20 mL) with different ratios (DI water : ethylene glycol is 1:0, 1:3, 1:1, 3:1, or 1:0), then heated and stirred for 10 min at 80 °C respectively. Then the Copper (II) acetate monohydrate solution was rapidly added to the NA solution with fast stirring at 1000 rpm. After intense stirring for 10 min, the solution was centrifuged at 7000 rpm followed by washing with 60 °C DI water and absolute ethanol for three times respectively to obtain the CuNA. At last, the blue powders were yielded by the freeze-drying method and stored at 4 °C for further studies.

### CuNA loading with bFGF

The 760 µg of bFGF was dissolved in 10 mL PBS, followed by the addition of CuNA powders and mixed well in a shaking bed for 1 h at 37 °C. Finally, the solution was centrifuged at 7000 rpm followed by the freeze-drying method to obtain the CuNA-bFGF powders. To determine the loading efficiency of CuNA, we measured the residual concentration of bFGF in the total supernatant by BCA protein kit and calculated the residual bFGF amount. Then we calculated the loading efficiency of bFGF using (the mass of bFGF - the mass of residual bFGF)/(the mass of CuNA for the mass of bFGF).

### Preparation of composite hydrogel

The 0.6 g of GelMA was dissolved in 10 mL PBS and stirred for 1 h at 37 °C. Then the powders of CuNA or CuNA-bFGF were mixed with the solution at concentration of 0, 3, 5, 10 and 20 wt%. Next, the above mixtures placed into a polytetrafluoroethylene (PTFE) mold with 10 mm inner diameter were exposed to UV light for 2 h and stored at 4 °C overnight. After removing the mold, composite hydrogels (CuNA@GelMA and CuNA-bFGF@GelMA) with different concentrations of CuNA or CuNA/bFGF were obtained.

### Physicochemical characterization of CuNA

NA and CuNA particles were analyzed via FTIR (Thermo Scientific Nicolet iS5, USA). X-ray diffraction (XRD, Rigaku Ultima IV, Japan) was employed to analysis the phase information of CuNA. The morphology and distribution of CuNA dissolved in absolute ethanol was viewed on scanning electron microscopy with the accessory EDS system (SEM, FEI, Nova 450, USA). The morphological structure and the surface chemistry of CuNA were observed by TEM (FEI, Talos F200S, USA). Protein binding was studied by UV–Vis spectroscopy (Thermo Scientific, USA). The zeta-potential and size distribution of CuNA in PBS (pH = 7.4) were measured by DLS (Malvern Zetasizer Nano ZS90, U.K.). The zeta potential also confirmed the effective binding of bFGF to the CuNA particles.

### Morphology observation of composite hydrogels

Wet GelMA and CuNA-bFGF@GelMA samples were frozen at - 80 °C and then lyophilized. The structure details and elemental mapping of GelMA and CuNA-bFGF@GelMA hydrogels were imaged by SEM. The surface chemistry of composite hydrogel was analyzed via XPS (Thermo Scientific, USA) .

### Release behavior of bioactive ingredients from composite hydrogels

CuNA-bFGF and CuNA-bFGF@GelMA were incubated into 10 mL PBS at a shaking speed of 200 rpm at 37 °C. At predetermined time points, 500 µL of release media was taken out and centrifuged at 7000 rpm. The supernatant was collected for content measurement and the release media were replenished with an equal volume of fresh media at 37 °C. The mount of bFGF was measured by BCA protein kit, copper was measured via inductively coupled plasma mass spectrometry (ICP-MS, PerkinElmer, USA), and NA was measured via UV–Vis spectroscopy (Thermo Scientific, USA).

### Mechanical performance of composite hydrogels

The mechanical properties of the GelMA composites were evaluated by compression test by a universal machine tester (HY-940FS, Shanghai, China). The samples for the compression tests were prepared in 10 mm inner diameter cylinder molds. Compressive tests were conducted at a rate of 5 mm/min to the strain of 90%. Compressive young’s modulus was determined from the slope of the strain-stress curve between 10 and 15% strain. The cyclic compression tests were performed with ten cycles up to 50% strain followed the speed of 5 mm/min to characterize the mechanical properties of GelMA composites. Each sample was tested in quadruplicate.

### Swelling behavior and biodegradation test

The swelling capacity of GelMA and GelMA composites was determined by swelling them in PBS at 37°. At predetermined time points, samples were taken out and wiped off the water on the surface with filter paper and weighed. The swelling ratio (SR) was obtained according to the formula:

$${\text{SR}}\left( \% \right) = \left[ {\left( {{\text{Wt}}-{\text{Wo}}} \right)/{\text{Wo}}} \right] \times 100,$$

where Wt was the weight of the wet samples at predetermined intervals, and Wo was the initial weight of the samples. Biodegradation tests of GelMA composites in vitro were performed by collagenase digestion. Samples were prepared as described above and freeze-dried. Then, samples were weighed and added to centrifuge tubes with 5 mL PBS containing 1 U/mL collagenase II at 37 °C. At the predetermined time points, samples were took out and dried in an oven at 50 °C. The mass residual was calculated by comparing the weight of digested samples at predetermined time intervals (Wt) with their original weight (Wo) using the following equation:

$${\text{Mass residual}}\left( \% \right) = \left( {{\text{Wt}}/{\text{Wo}}} \right) \times 100.$$

### In vitro antibacterial activity evaluation of composite hydrogels

Gram-negative bacteria of *Escherichia coli* (*E. coli*, ATCC 8739) and Gram-positive bacteria of *Staphylococcus aureus* (*S. aureus*, ATCC 6538) were selected for evaluating the antibacterial ability of CuNA crossed-linked GelMAs. The agar disc diffusion method and MTT method [55] were performed to evaluate the antibacterial activities of CuNA or CuNA crossed-linked GelMA. In agar disc-diffusion method, GelMA composites (10 mm in diameter and 2 mm in height) were prepared and UV-sterilized as described above. Pure GelMA was used as the positive control. The suspension of *E. coli* and *S. aureus* were inoculated with sterile pipette tips on LB agar plates respectively and incubated at 37 °C for 24 h. The resulting zones of inhibition will be uniformly circular if inhibitory concentrations were reached. The inhibition zones of all samples were measured and results were photographed for further calculated.

In MTT assay, CuNA powder was diluted with LB medium into different concentrations and a 1.0 × 10^6^ CFU/mL suspension was obtained after mixing with the bacterial suspension. The bacteria treated with LB without CuNA served as the control group. After 16 h of incubation at 37 °C, MTT stock solution was added to LB mediums mentioned above, and the mixtures were incubated at 37 °C for 4 h. Then the formazan crystals were dissolved with DMSO at room temperature, and the OD value was measured at 570 nm using a spectrophotometer (Evolution UV201, Thermo Fisher Scientific, USA).

### Cell culture

The NIH/3T3 and HUVEC cell lines were obtained from Cell Bank of the Chinese Academy of Sciences (Shanghai, China) and cultured in DMEM medium with 10% fetal bovine serum and 1% penicillin-streptomycin in a CO_2_ incubator with 5% level at 37 °C. Samples including GelMA, CuNA@GelMA, and CuNA-bFGF@GelMA prepared for making ionic extraction were sterilized by 75% ethanol for 2 h and washed three times with PBS (15 min/time). Ionic extraction of these samples were prepared according to International Standard Organization (ISO/EN) 10993-5 by directly immersing samples into DMEM with 10% FBS at 37 °C in an incubator with 5% CO_2_ for 24 h.

### Cell cytotoxicity and proliferation assessment

Two types of cells were seeded in 12-well plates with a density of 1 × 10^4^/well and cultured in the complete medium with different ionic extraction mentioned above. Cell cytotoxicity was determined by a live/dead cell assay kit according to the manufacturer’s instruction after incubation for 24 h. Cell viability was further assessed by a CCK-8 method. Briefly, two types of cells were seeded in 96-well plates with a density of 1 × 10^4^/well and cultured with different ionic extraction mentioned above in the CO_2_ incubator with 5% level at 37 °C for 1 and 2 days. Cells cultured in the complete medium without extraction were set as the control group. At predetermined time points, the medium was removed and washed two times with PBS, and then 100 µL of serum-free medium with 10 µL of CCK-8 solution was added to each well. After incubation in a CO_2_ incubator at 37 °C for 2 h, absorbance was detected at 450 nm using a microplate reader (Thermo Fisher, USA).

For the proliferation test, the two types of cells were seeded at an initial density of 5 × 10^3^ cells/well in 96-well plates with ionic extraction at 37 °C in a CO_2_ incubator with 5% level. On day 1, 3, 5 and 7, the CCK-8 method was performed to assess the cell viability. The cell viability was determined according to the equation:

$${\text{Cell viability}}\left( \% \right) = \left( {{\text{ODs}}/{\text{ODc}}} \right) \times 100,$$

where ODs, ODc indicate the absorbance of samples and control group respectively. To ensure the validity of experimental data, all the samples were assayed in triplicate.

### Cell attachment and spreading behavior studies

Before cell attachment, GelMA and GelMA composite precursors were placed in a 12-well plate with a thickness of 2 mm followed by exposure to UV light for 2 min. Then samples were washed with PBS (pH = 7.4) three times followed by exposure to UV light for 2 h. Then two types of cells were seeded at an initial density of 1 × 10^4^ cells/well in 12-well plates and cultured with complete medium. After incubation for 2 days, the cells were rinsed with PBS and fixed with 4% paraformaldehyde for 10 min. After permeabilization by 0.2% Triton X-100 solution for 2 min, cells were incubated with rhodamine–phalloidin and anti-vinculin solutions for 45 min and followed by incubation with DAPI for 5 min. The fluorescent images were taken using a fluorescence microscope (Leica, Germany).

### Migration and tubule formation assay

A transwell assay was performed to evaluate the migration ability of HUVEC and NIH/3T3 in the ionic extraction. Two types of cells were diluted with serum-free medium to 1.0 × 10^5^ cells/mL, then added 200 µL in the upper chamber of a 24-well transwell plate (3422, Corning, USA) and adding 500 µL of ionic extraction to the lower chamber. As a control, 500 µL complete medium was added to the lower chamber. After incubation at 37 °C for 24 h, cells on the upper chamber were scraped off using a dry cotton swab. Cells migrated to the lower surface were washed with PBS, fixed with 4% paraformaldehyde, and stained with 0.1% crystal violet. The migrated cells were imaged under a microscope (Leica, Germany). All the samples were assayed in triplicate and each sample was observed with 3 random zones to count the migration cells.

The angiogenic ability of HUVEC in different ionic extraction was evaluated by seeding cells on the Matrigel matrix. Briefly, the matrix was spread in a pre-cooled 96-well plate using precooled tips and solidified at 37 °C for 1 h. Then 100 µL HUVEC suspension with 2.0 × 10^4^ cells/mL was seeded on the matrix and incubated for 4 h. The total segment length was measured by ImageJ with the Angiogenesis Analyzer plugin [56] to assess the tubule formation ability.

### Live/dead assay

The effect of ionic extraction of GelMA composites on cells was tested by a live/dead assay. HUVEC and NIH/3T3 were incubated in the complete medium at an initial density of 1 × 10^4^ per well in a 96-well plate for 24 h. Then the medium was removed, and ionic extraction of CuNA@GelMA and CuNA-bFGF@GelMA was added to each well. After incubated for another 24 h, cells were washed with PBS three times followed by adding 100 µL staining solution per well. After incubated at 37 °C for 30 min, two types of cells were imaged under a fluorescence microscope (Leica, Germany).

### Full-thickness skin defect model and treatment

Female Sprague-Dawley (SD) rats (B & K Universal Ltd., China) were prepared for building the full-thickness skin defect model by surgical incision. Briefly, after anaesthetization with 3% sodium pentobarbital (intraperitoneal, 30 mg/kg), the dorsum of rats was depilated with a shaver and cleaned with 75% alcohol. Then two full-thickness excisional wounds (10 mm × 10 mm) were made on both sides of each rat. The defects were covered with GelMA, 5% CuNA@GelMA and 5% CuNA-bFGF@GelMA prepared as mentioned above and fixed with medical tapes (3 M, USA). The wounds treated with only medical tapes were setted as the negative control group (Blank). After sacrificed at day 3, 7 and 14, the wound and normal adjacent skin were excised and then fixed in 4% paraformaldehyde for further histological and immunohistochemical analysis.

### Wound closure measurement

The wounds were observed with a digital camera after 3, 5, 7, 11 and 14 days and the wound area was measured by ImageJ software. The percentage of wound closure was defined with the equation:

$${\text{Wound closure}}\left( \% \right) = \left( {1 - P/I} \right) \times 100,$$

where I is the original wound area and P is the wound area at a given time point. For statistical analysis, three samples were analyzed for per treated group.

### Histological, immunofluorescence, and immunohistochemical evaluation

Tissues excised and fixed in the paraformaldehyde solution were dehydrated and embedded in paraffin and then sectioned into 5 μm and mounted on slides for staining. Thereafter, sections were stained with hematoxylin and eosin (H&E, servicebio, China) and Masson’s trichrome staining Kit (servicebio, China) following the manufacturer’s instructions. Subsequently, sections were stained with anti-CD31 (abcam, USA), anti-α-SAM antibodies (abcam, USA) and DAPI respectively. To identify elastic fibers, Weigert’s elastic staining was used to stain the sections. The immunostaining of IL-6 (1:600 dilutions, rabbit polyclonal, servicebio, China), CD34 (1:600 dilutions, rabbit polyclonal, servicebio, China), Ki67 (1:600, rabbit polyclonal, servicebio, China) were performed following the manufacturer’s instructions. The images were captured with a digital microscope (Leica, USA).

### Statistical analysis

Data are expressed as mean ± standard deviation (SD). The significance between the groups was analyzed by student’s *t* test and one-way analysis of variance (ANOVA) using SPSS software (version 25.0, IBM). The statistical significance was set at *p < 0.05, ** < 0.01, *** <0.001.

## Supplementary Information

**Additional file 1: Figure S1.** TEM images of CuNA preparedin DI water and ethylene glycol with different ratio. (a) DI water. (b) DI water:ethylene glycol = 3:1. (c) DI water:ethylene glycol = 1:1. (d) DI water:ethyleneglycol = 1:3. **Figure S2.** (a-c) Three parallel samples of CuNA andCuNA-bFGF dissolved in PBS for zeta potential test. **Figure S3.** SEM and elemental mapping images of CuNA-bFGF@GelMA. Scale bar is 200 μm. **Figure S4.** XPS analysis of the elemental composition on composite hydrogel. **Figure S5.** The release profile of copper from CuNA-bFGF and composite hydrogel at first hour. **Figure S6.** The release profile of NA from CuNA-bFGF and compositehydrogel (a). The release profile of NA of 72 h; (b). The release profile of NA at first hour. **Figure S7.** Didital images of pure CuNA disks during the antibacterial test against *E. coli* (a) and*S. aureus* (b). Scale bar is 10 mm. **Figure S8.** Immunohistochemical staining for CD34 on day 3 and 7. Scale bar is 100 μm. (The newly formed vessels, black arrow). **Figure S9.** Masson’s trichromatic staining for normal tissues.

## Data Availability

All data generated or analyzed during this study are included in this published article.

## Abbreviations

bFGF: Basic fibroblast growth factor
ECM: Extracellular matrix
MOF: Metal–organic frameworks
TEM: Transmission electron microscopy
XRD: X-ray diffraction spectra
FTIR: Fourier Transform Infrared Spectrum
DLS: Dynamic light scattering
ICP-MS: Inductively coupled plasma mass spectrometry
XPS: X-ray Photoelectron Spectroscopy
HUVEC: Human umbilical vein endothelial cells
MTT: 3-(4,5-Dimethylthiazol-2-yl)-2,5-diphenyltetrazolium bromide
CCK-8: Cell counting kit-8
SD: Standard deviation
PBS: Phosphate buffered saline
BCA: Bicinchoninic acid
