# Supplementary material for: Donut-like MOFs of copper/nicotinic acid and composite hydrogels with superior bioactivity for rh-bFGF delivering and skin wound healing
Source: J Nanobiotechnology. 2021 Sep 9;19:275. doi: 10.1186/s12951-021-01014-z (PMC8427876; doi:10.1186/s12951-021-01014-z)
Supplement: Supplementary file 1 — Additional file 1: Figure S1. TEM images of CuNA preparedin DI water and ethylene glycol with different ratio. (a) DI water. (b) DI water:ethylene glycol = 3:1. (c) DI water:ethylene glycol = 1:1. (d) DI water:ethyleneglycol = 1:3. Figure S2. (a-c) Three parallel samples of CuNA andCuNA-bFGF dissolved in PBS for zeta potential test. Figure S3. SEM and elemental mapping images of CuNA-bFGF@GelMA. Scale bar is 200 μm. Figure S4. XPS analysis of the elemental composition on composite hydrogel. Figure S5. The release profile of copper from CuNA-bFGF and composite hydrogel at first hour. Figure S6. The release profile of NA from CuNA-bFGF and compositehydrogel (a). The release profile of NA of 72 h; (b). The release profile of NA at first hour. Figure S7. Didital images of pure CuNA disks during the antibacterial test against E. coli (a) andS. aureus (b). Scale bar is 10 mm. Figure S8. Immunohistochemical staining for CD34 on day 3 and 7. Scale bar is 100 μm. (The newly formed vessels, black arrow). Figure S9. Masson’s trichromatic staining for normal tissues. [file 12951_2021_1014_MOESM1_ESM.docx]

**Additional Information**

**Donut-Like MOFs of Copper/Nicotinic Acid and Composite Hydrogels with Superior Bioactivity for rh-bFGF Delivering and Skin Wound Healing**

Tian-Long Wang^1#^, Zi-Fei Zhou^1,2,#^, Jun-Feng Liu^1^, Xiao-Dong Hou^1^, Zhi Zhou^1^, Yun-Lu Dai^1^, Zhi-Yong Hou^3*^, Feng Chen^1*^ and Long-Po Zheng^1,2*^

^1^ Department of Orthopedics, Shanghai Tenth People’s Hospital, Tongji University School of Medicine, Shanghai 200072, China

^2^ Shanghai Trauma Emergency Center, Shanghai 200072, China

^3^ Department of Orthopaedic Surgery, Third Hospital of Hebei Medical University, Shijiazhuang 050051, China

^4^ Cancer Centre and Institute of Translational Medicine, Faculty of Health Sciences, University of Macau, Macau SAR, 999078, China

# These authors contributed equally to this work.

***** Corresponding author:

E-mail: drzyhou@gmail.com (Zhi-Yong Hou); fchen@tongji.edu.cn (Feng Chen); dr.zheng@tongji.edu.cn (Long-Po Zheng)


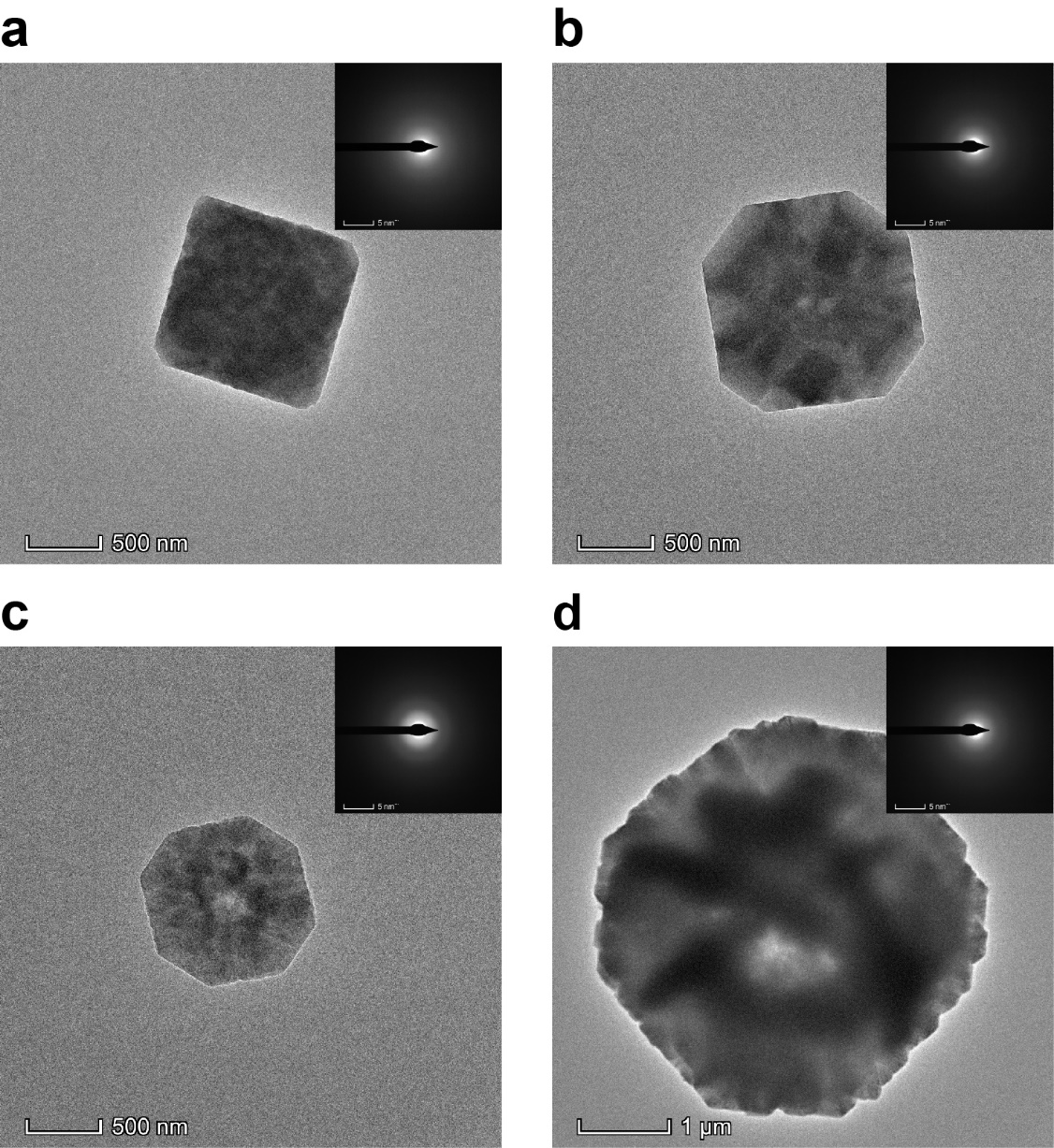


**Figure S1** TEM images of CuNA prepared in DI water and ethylene glycol with different ratio. (a) DI water. (b) DI water : ethylene glycol = 3 : 1. (c) DI water : ethylene glycol = 1 : 1. (d) DI water : ethylene glycol = 1 : 3.


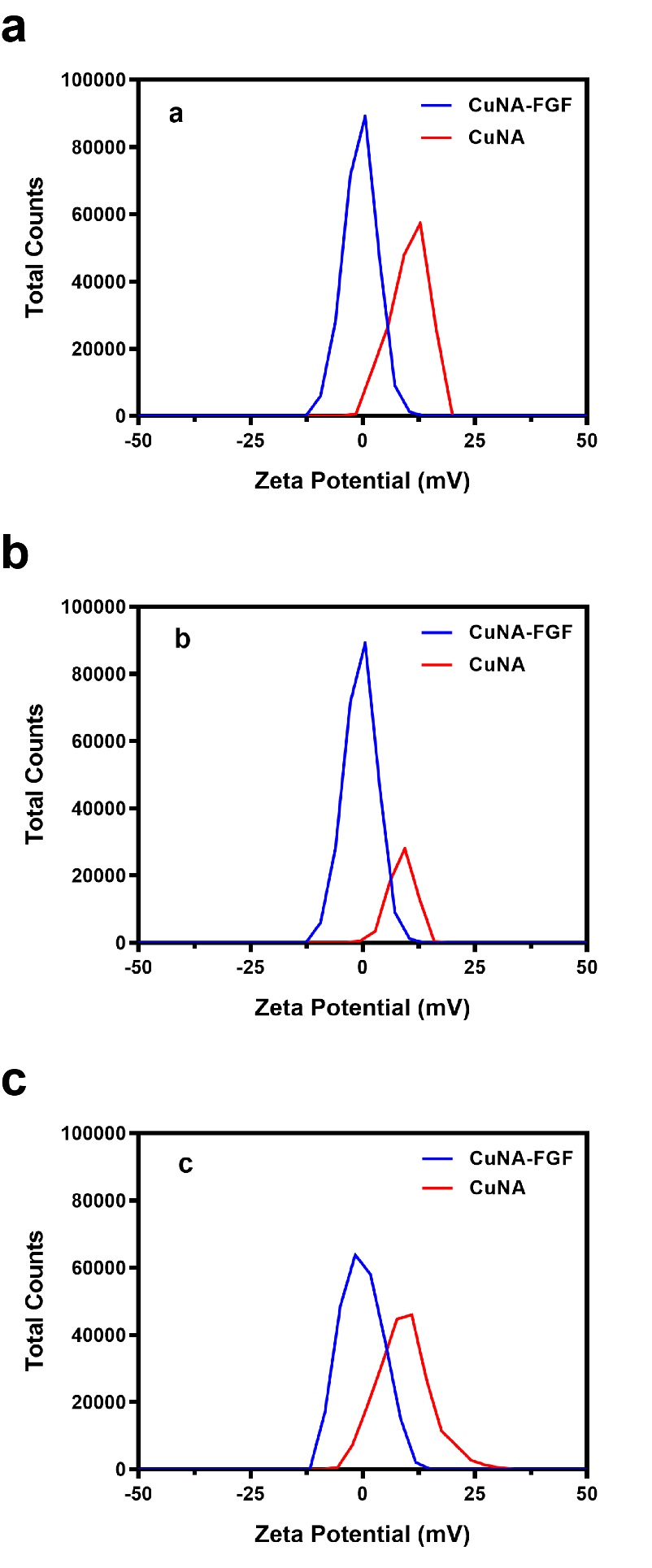


**Figure S2** (a-c) Three parallel samples of CuNA and CuNA-bFGF dissolved in PBS for zeta potential test.


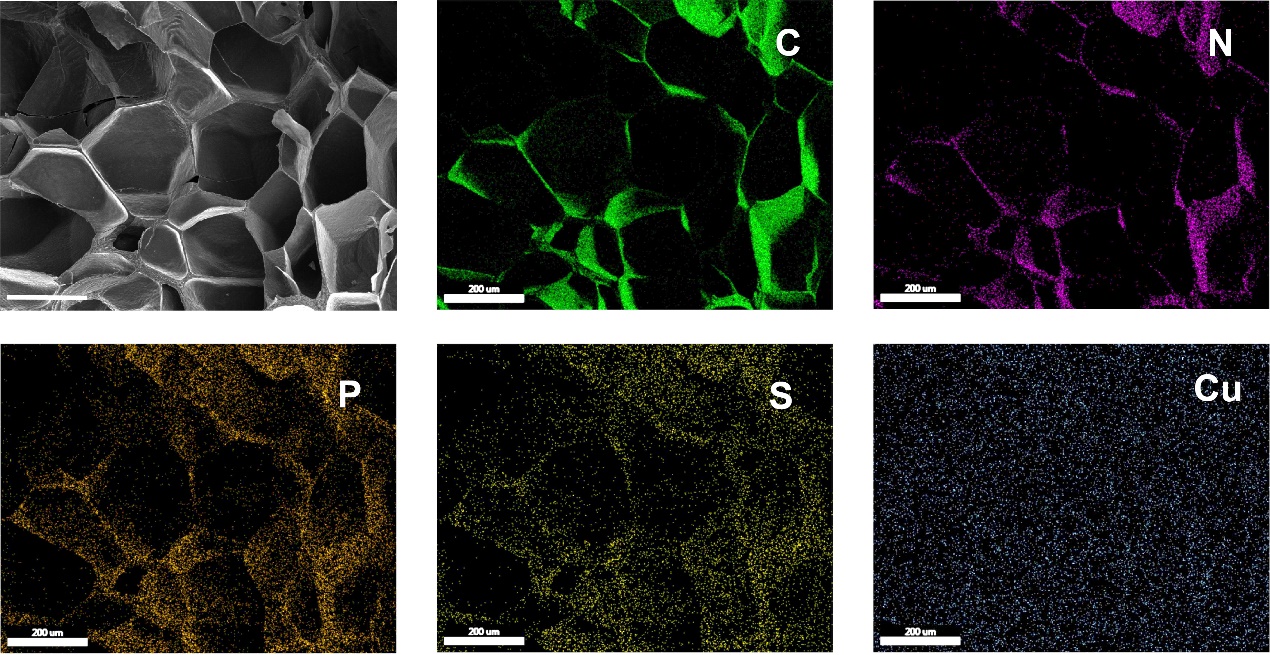


**Figure S3** SEM and elemental mapping images of CuNA-bFGF@GelMA. Scar bar is 200 μm.

**
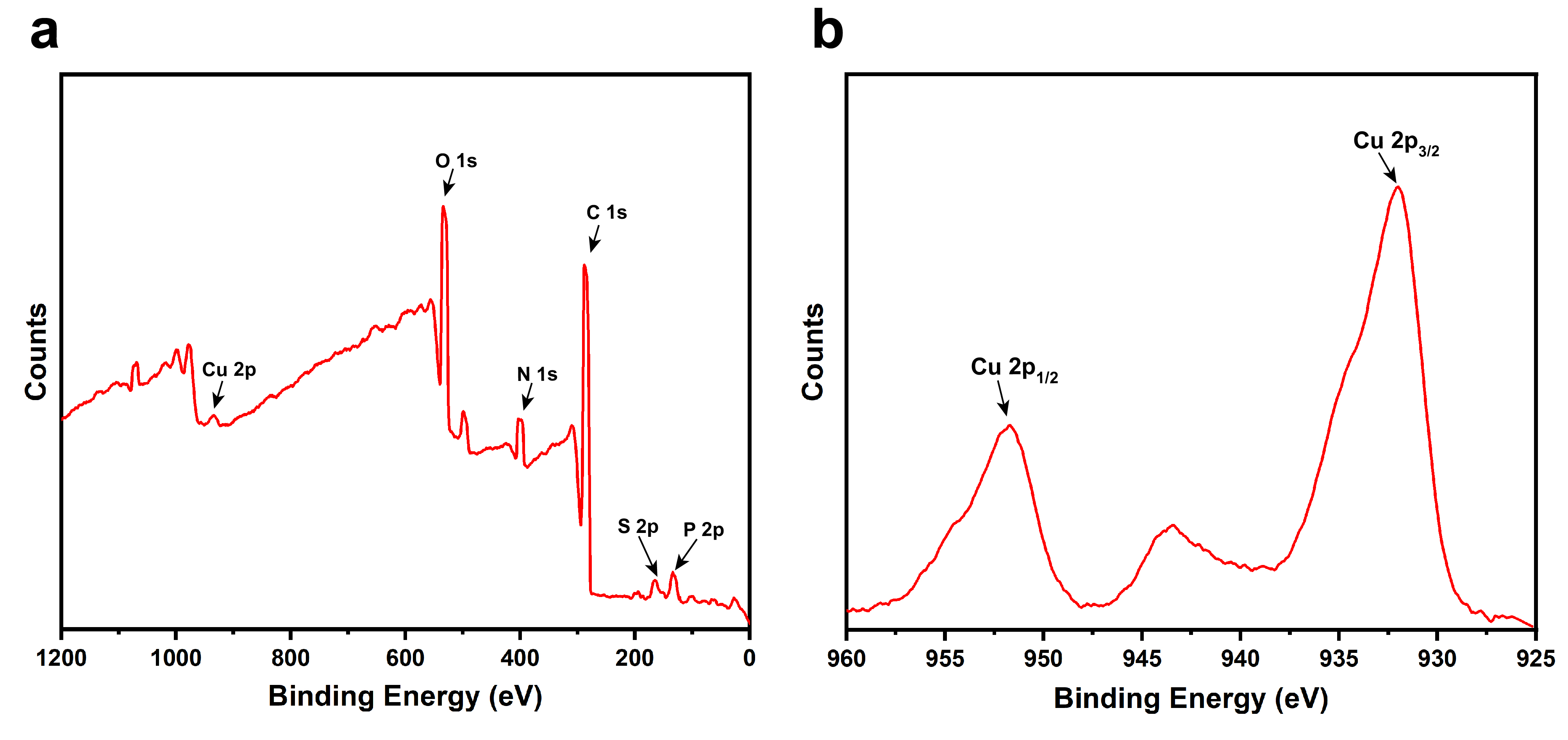
**

**Figure S4** XPS analysis of the elemental composition on composite hydrogel.

**
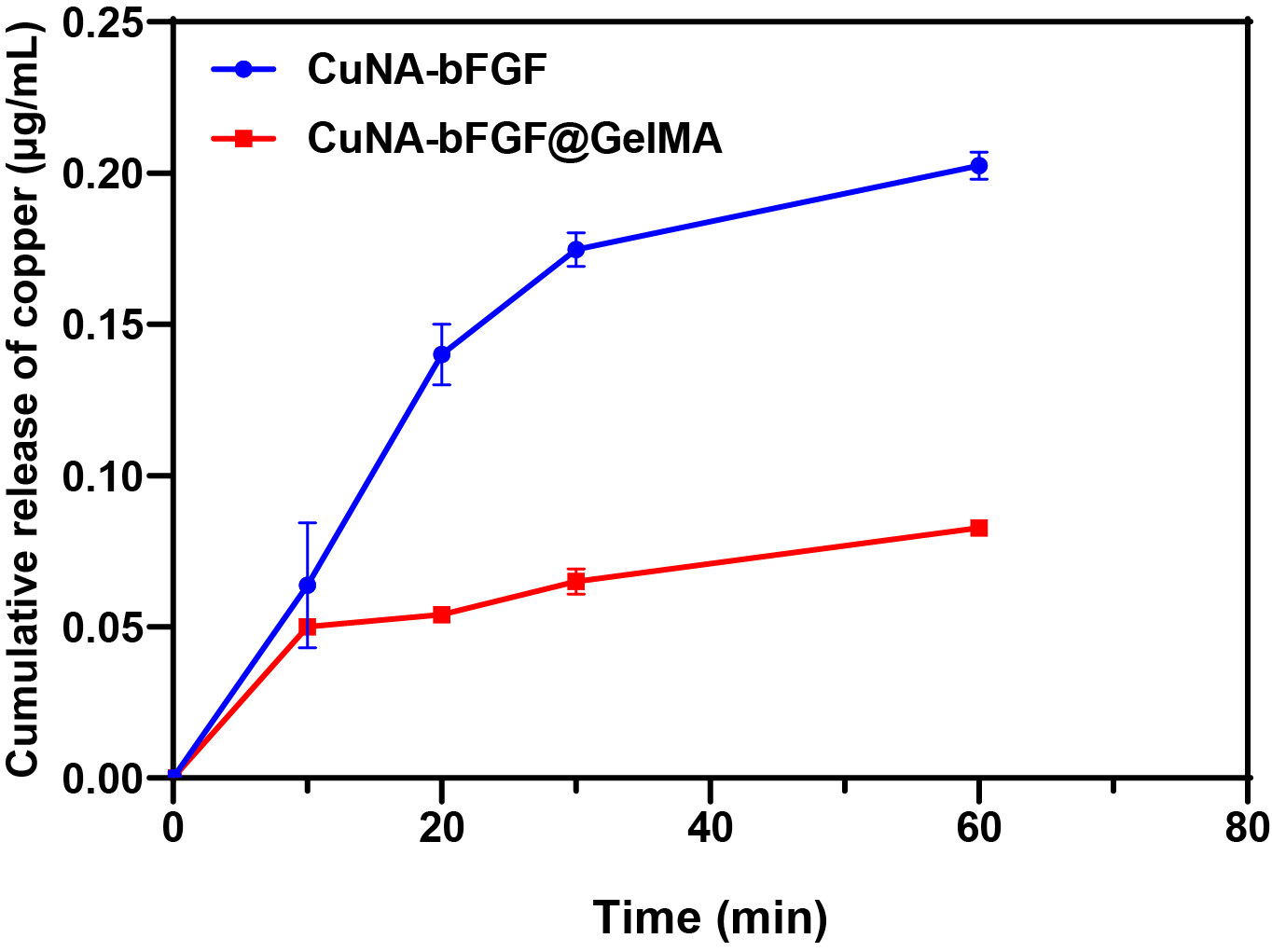
**

**Figure S5** The release profile of copper from CuNA-bFGF and composite hydrogel at first hour.

**
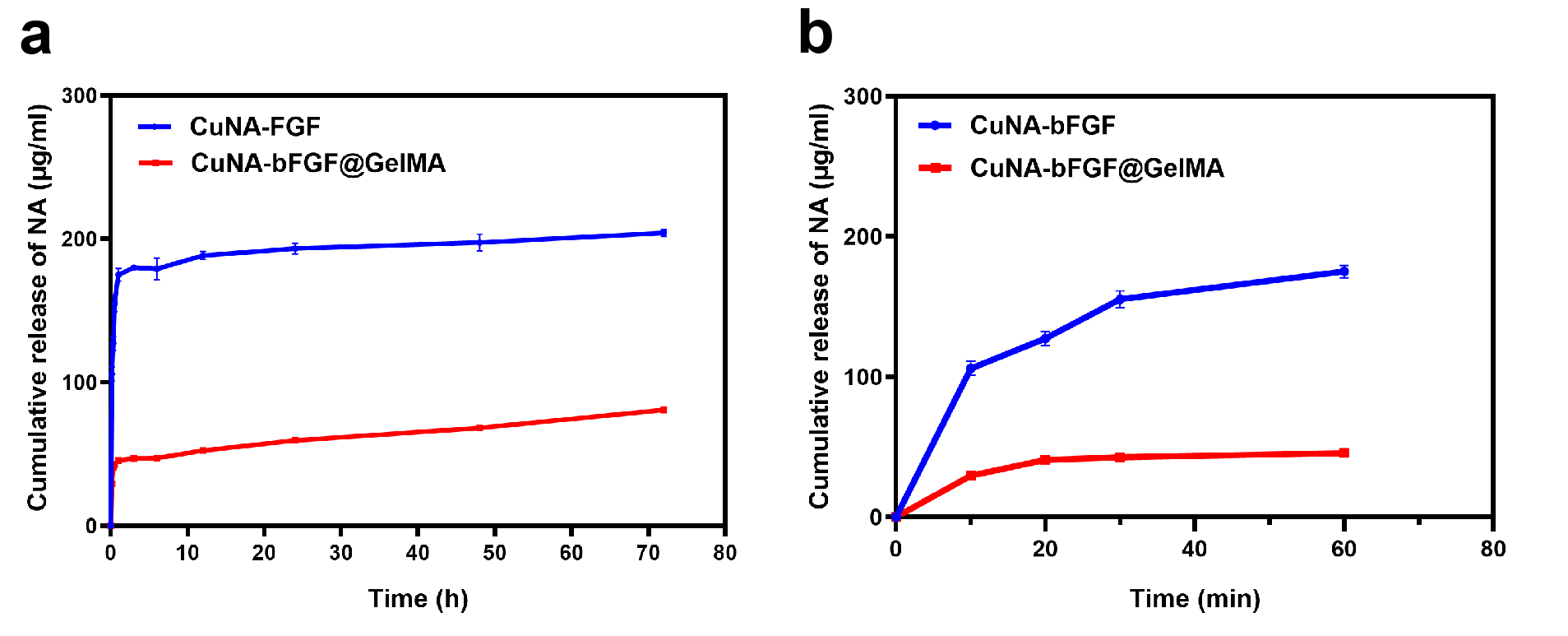
**

**Figure S6** The release profile of NA from CuNA-bFGF and composite hydrogel (a). The release profile of NA of 72 h; (b). The release profile of NA at first hour.


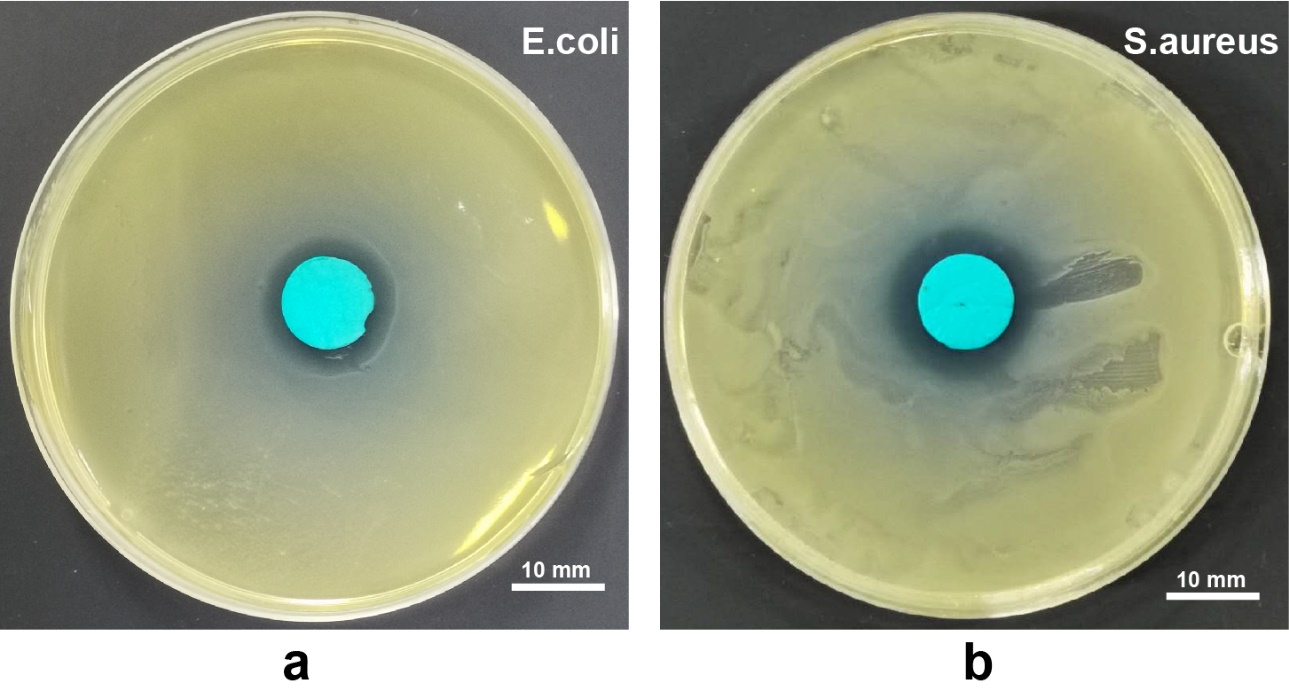


**Figure S7** Didital images of pure CuNA disks during the antibacterial test against E. coli (a) and S. aureus (b). Scar bar is 10 mm.


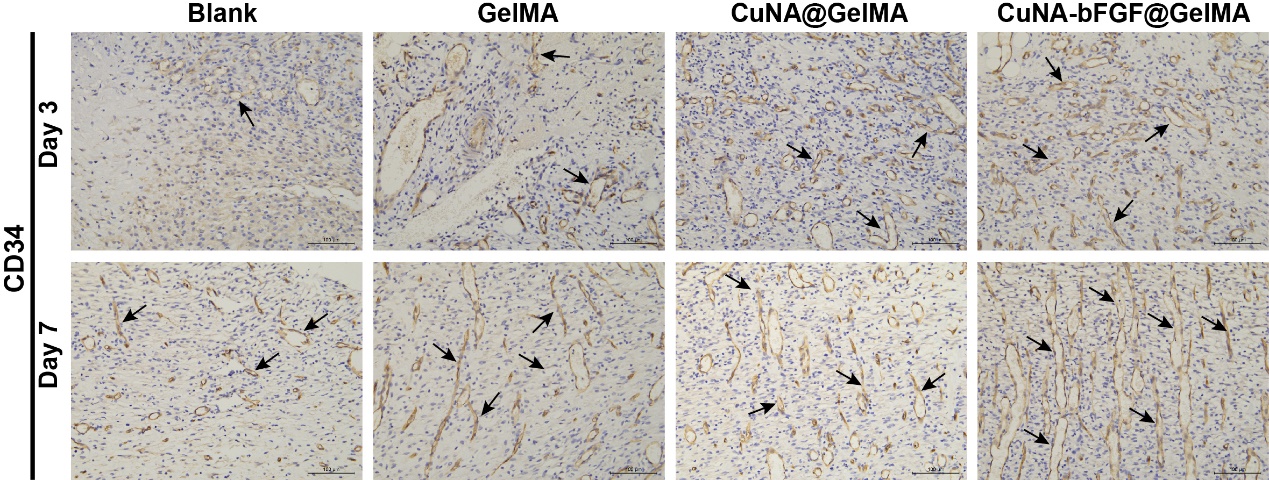


**Figure S8** Immunohistochemical staining for CD34 on day 3 and 7. Scar bar is 100 μm. (The newly formed vessels, black arrow)


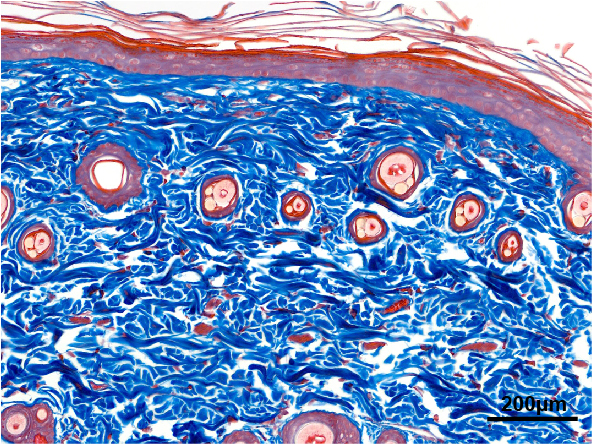


**Figure S9** Masson’s trichromatic staining for normal tissues**.**
